# Extracellular matrix-growth factor signalling drives the oncogenic mir-125b-2/UCK2 axis in hepatocellular carcinoma

**DOI:** 10.1186/s41065-026-00673-y

**Published:** 2026-04-02

**Authors:** YunFeng Zhao, HaoXin Shen, Yibo Sun, JinKun Song, Chen GuangSun, JinXiu Li, HongYu Wang, YinSen Song

**Affiliations:** 1 Department of Liver Transplant, Zhengzhou People’s Hospital, Zhengzhou, 450000 Henan China; 2 Department of Critical Care Medicine, Zhengzhou People’s Hospital, Zhengzhou, 450000 Henan China; 3 Key Laboratory of Organ Transplantation, Zhengzhou People’s Hospital, Zhengzhou, 450000 Henan China

**Keywords:** Hepatocellular carcinoma, miRNA/mRNA axis, Extracellular matrix signaling, Growth-factor circuits, Prognostic biomarkers

## Abstract

**Background:**

Hepatocellular carcinoma (HCC) is characterized by coordinated transcriptional and post-transcriptional dysregulation. We sought to identify clinically relevant miRNA–mRNA regulatory axes with cross-cohort, multi-layer validation.

**Methods:**

Tumor–normal differential expression was integrated across TCGA-LIHC and independent microarray cohorts. Predicted miRNA targets were filtered by inverse-direction overlap with consensus DEGs and evaluated for activity–abundance coherence and survival relevance. An eight-feature axis (four miRNAs and four target-set activities) was modeled using penalized Cox regression with cross-validation and externally validated.

**Results:**

Four recurrent miRNAs were identified (miR-125b-2 downregulated; miR-21, miR-221, miR-9-1 upregulated). miR-125b-2 showed the strongest inverse coherence with its target set (*ρ* = −0.41, *P* < 1 × 10⁻¹²). The composite axis stratified TCGA overall survival (log-rank *P* < 0.0001) and validated in GSE31384 (HR = 1.62, 95% CI 1.15–2.28; C-index = 0.66). Seven targets met FDR ≤ 1%, with UCK2 exhibiting the strongest adverse association (HR = 2.78, *P* = 2.7 × 10⁻⁹). UCK2 was overexpressed, hypomethylated, enriched in epithelial compartments, and linked to proliferative and ECM/growth-factor signaling programs. Functional assays demonstrated that UCK2 knockdown suppressed proliferation, clonogenicity, and migration, whereas overexpression enhanced these phenotypes.

**Conclusions:**

The miR-125b-2/UCK2 axis defines a metabolically driven, epithelial proliferative program coupled to ECM/GF signaling and represents a validated prognostic and biologically actionable node in HCC.

**Graphical Abstract:**

**Figure:**
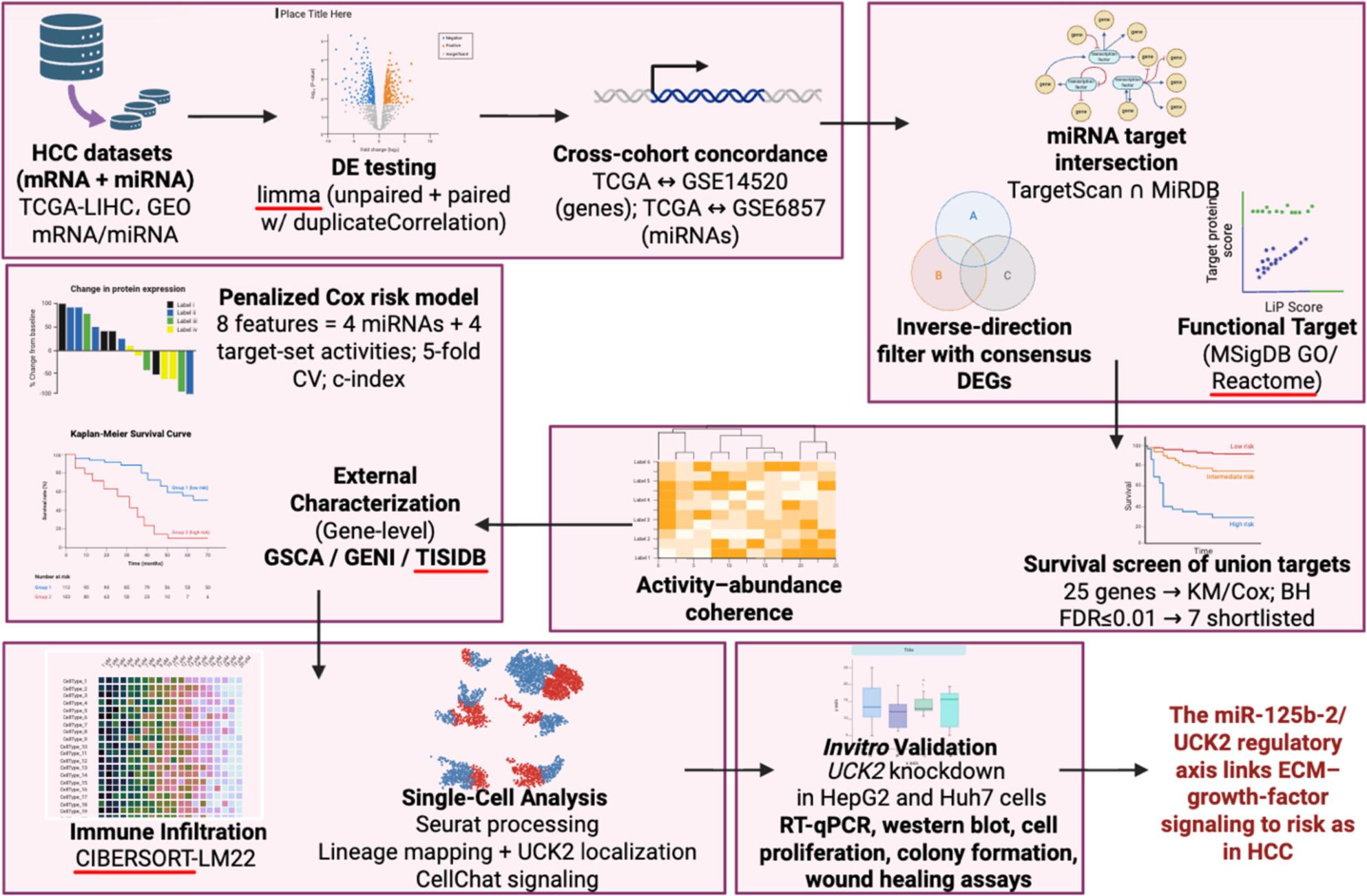
Bulk and single-cell multi-omics were integrated to define prognostic miRNA–mRNA axes in hepatocellular carcinoma (HCC). TCGA-LIHC and GEO (GSE14520/GSE6857) underwent limma differential expression, cross-cohort concordance and miRNA target intersection filtered by inverse-direction DEGs and functionally profiled with MSigDB (GO/Reactome). Matched tumors enabled activity–abundance coherence testing; an 8-feature penalized-Cox model (4 miRNAs + 4 target-set activities) stratified TCGA overall survival and validated externally (GSE31384). A survival screen of 25 union targets shortlisted seven genes (risk: UCK2; protective: CPEB3, PIK3R1, SUN2, CLDN14, PDK4, OGDHL) and gene-level characterization used GSCA/GENI/TISIDB. CIBERSORT-LM22 deconvolution and Seurat/CellChat placed UCK2 in epithelial lineages and revealed ECM/growth-factor crosstalk.

**Supplementary Information:**

The online version contains supplementary material available at 10.1186/s41065-026-00673-y.

## Introduction

Hepatocellular carcinoma (HCC) remains a leading cause of cancer mortality worldwide and commonly arises by setting of chronic liver disease. Despite advances in surveillance and locoregional therapy, most patients present with advanced disease and molecularly informed biomarkers are still needed to refine risk stratification and to guide treatment strategies [1, 2]. Transcriptional programs involving cell-cycle progression, metabolic rewiring and microenvironmental remodeling are recurrent features of HCC [3, 4]. Several studies have been performed to identify novel biomarkers associated with higher mRNA expression and prognosis in HCC including PRDX6 and KIF5B [5–7]. Post-transcriptional controls mediated by microRNAs (miRNAs) have also a role in progression which collectively modulate mRNA stability and translation and can consolidate oncogenic or tumor-suppressive states [8].

Several miRNAs have been implicated in several cancers biology and prognosis. miR-21 is frequently up-regulated in cancers and has been linked to pro-proliferative and pro-invasive phenotypes through targets in PTEN/PI3K signaling and extracellular-matrix (ECM) regulation [9]. miR-221 is similarly reported as an oncomiR with connections to cell-cycle control and receptor tyrosine kinase (RTK) pathways [10]. In contrast, miR-125b is widely described as tumor-suppressive, with repression observed across multiple tumor types including HCC [11, 12]. The extent to which these miRNAs act through coherent, DEG-supported target sets and how such axes aggregate into prognostic signals remains incompletely resolved.

Several studies have also established members of the miR-125 family as tumor suppressors in HCC. Reduced miR-125b expression has been associated with enhanced proliferation, invasion, and poor prognosis, partly through regulation of oncogenic signaling components including PI3K/AKT and receptor tyrosine kinase pathways [11]. However, prior investigations have largely focused on individual downstream targets or isolated signaling pathways, without defining a systems-level regulatory axis integrating miR-125b dysregulation with metabolic drivers and extracellular matrix/growth factor (ECM/GF) signaling networks. In particular, the potential linkage between miR-125b-2 suppression, UCK2-mediated nucleotide metabolism, and matrix-coupled intercellular communication has not been systematically explored in HCC.

Integration of bulk RNA-seq and miRNA-seq enables rigorous tumor–normal differential expression and provides the scale needed for survival modeling. However, external validation is essential to mitigate cohort-specific artifacts. Furthermore, annotation of axes within the tissue ecosystem benefits from single-cell RNA-seq (scRNA-seq) which resolves lineage specificity and allows inference of cell–cell communication via ligand–receptor frameworks such as CellChat [13]. Complementary portals including GSCA [14], GENI [15] and TISIDB [16] help position candidate genes within tumor–normal contrasts, methylation status, pathway hallmarks, immune correlates and druggability landscapes.

In this study, bulk differential expression was performed for TCGA-LIHC mRNA and miRNA together with discovery microarrays (GSE14520, GSE6857). Predicted targets from TargetScan and MiRDB were intersected and constrained to inverse-direction overlaps with consensus Differentially Expressed Genes (DEGs) to construct a miRNA–mRNA network. The four miRNAs (miRNAs—miR-125b-2, miR-21, miR-221 and miR-9-1) were emerged from a direction-consistent cross-cohort differential expression framework (TCGA paired/unpaired and GSE6857) which were then selected for coherence testing, survival modeling and single-cell contextualization.

Functional enrichment used MSigDB GO and Reactome categories with relaxed thresholds to accommodate small gene sets while retaining biological interpretability. Coherence between miRNA abundance and the activity of its supported targets was quantified in matched TCGA tumors. An eight-feature “axes” matrix comprising four miRNA abundances and four target-set activities was evaluated using penalized Cox regression under nested cross-validation, compared against clinical-only models and externally validated in GSE31384 using a locked linear predictor. Survival screening of union targets used Kaplan–Meier and Cox models with False Discovery Rate (FDR) control. *UCK2* and other prioritized targets were then characterized using GSCA (expression, methylation, stage), GENI (Hallmark GSEA by gene–gene correlations), and TISIDB (immune correlates and drug–gene networks). Finally, scRNA-seq (GSE125449) and CellChat were used to localize candidate genes across liver/tumor lineages and to map intercellular signaling pathways. The aim was to resolve coherent miRNA–mRNA axes with prognostic value, to nominate mechanistically plausible pathways, and to place prioritized genes within epithelial lineage context and ECM/growth-factor signaling circuits relevant to HCC biology and prognosis.

## Methodology

### Bulk mRNA/miRNA differential expression

The Cancer Genome Atlas (TCGA)-LIHC RNA-seq and miRNA-seq datasets consisted of 371 tumors and 50 normals for mRNA, and 372 tumors and 50 normals for miRNA were used to analyze primary tumor and matched non-tumor liver tissues [17]. Lowly expressed features were removed using a CPM ≥ 1 and library sizes were normalized with edgeR and transformed with limma prior to modeling. This threshold is consistent with standard edgeR/limma preprocessing recommendations for RNA-seq data [18]. The same CPM filtering criterion was applied uniformly across TCGA-LIHC mRNA and miRNA datasets prior to normalization and modeling to ensure reproducibility. Microarray datasets (GSE14520 and GSE6857) were preprocessed according to platform-specific normalization procedures and therefore did not require CPM-based filtering. Two public microarray cohorts included GSE14520 [19] for mRNA expression and GSE6857 [20] for miRNA expression were also used as discovery datasets. Differential expression was assessed with linear models in *limma* package of R **(**RStudio 2025.05.1 + 513**)** [21]. For TCGA we fit both an unpaired design and a paired sensitivity analysis using duplicateCorrelation with patient ID as a blocking factor. *P*-values were adjusted using the Benjamini–Hochberg procedure (FDR ≤ 0.05). In addition to statistical significance, an effect-size filter was applied. For mRNA, a |log2FC| ≥ 1 (≥ 2-fold) threshold was used to prioritize genes. For miRNAs, a slightly lower fold-change cutoff (≥ 1.5-fold; |log2FC| ≥ log2(1.5)) was selected because miRNAs generally exhibit narrower dynamic ranges and can exert substantial regulatory effects with modest expression changes.

### miRNA target prediction and inverse-direction overlap with consensus DEGs

For each miRNAs, predicted mRNA targets were retrieved from TargetScan ( https://www.targetscan.org/vert_80/ ) [22] and MiRDB (https://mirdb.org/ ) [23]. Only genes present in both resources (TargetScan ∩ MiRDB) were retained and mapped to HGNC symbols. To shortlist the pairs supported by HCC, TargetScan ∩ MiRDB were intersected with DEGs (inverse) and up-regulated miRNAs target sets with the down meta-DEGs and the down-regulated miRNA’s target set (miR-125b-2) with the Up meta-DEGs. The resulting inverse-direction intersections were carried forward as candidate miRNA–mRNA regulatory axes and were validated via coherence between miRNA abundance and target-set activity in matched TCGA tumors and survival prioritization with Benjamini–Hochberg correction.

### Functional enrichment of DEG-supported miRNA candidate targets

Gene sets were obtained from MSigDB via msigdbr (Homo sapiens): C5 (GO: BP/CC/MF) and C2:CP (Reactome) [24]. Over-representation testing used clusterProfiler::enricher (hypergeometric test; pAdjustMethod = “BH”). The gene universe was set to all genes expressed after CPM filtering across the bulk cohorts and gene symbols were harmonized to HGNC. Because hypergeometric-based enrichment methods are sensitive to gene set size, no categories remained significant after multiple testing correction (FDR < 0.05), although biologically coherent trends were observed.

### Activity–abundance coherence

Matched TCGA-LIHC tumors profiled by both mRNA-seq and miRNA-seq were used. mRNA counts were converted to logCPM, mapped from Ensembl to HGNC symbols and symbols with duplicates were collapsed by the median. Within tumors, genes were row-z-scored and, for each candidate miRNA, a per-sample target-set activity was computed as the mean z across its supported targets. Coherence between miRNA abundance and target-set activity was quantified by Spearman’s rank correlation across tumors with both assays. For survival, KM-plotter (LIHC) was queried for each miRNA [25]. As mirPower does not distinguish precursors/arms, family-level entries were used (miR-21, miR-221, miR-125b, miR-9) with the default high/low split and univariable Cox/log-rank outputs.

### Machine-learning survival modeling

For TCGA-LIHC tumors profiled by both mRNA-seq and miRNA-seq, an eight-feature “axis” matrix was assembled comprising four miRNA abundances (logCPM for miR-21, miR-221, miR-125b-2, miR-9-1) and four target-set activities. Clinical covariates (age, sex, pathologic stage) were taken from GDC clinical data. After merging molecular and clinical data with overall-survival (OS) outcomes, 127 complete cases remained. Unsupervised structure of the eight molecular features was examined by principal component analysis and k-means clustering for K = 2–6, with average silhouette width used to assess separation. For supervised survival prediction, penalized Cox regression [26] was trained under a common 5-fold cross-validation (CV) split for three specifications i.e., clinical-only (age, sex, stage), axes-only (eight molecular features) and combined (clinical + axes). Within each training fold, the elastic-net mixing parameter was tuned by inner CV; performance on the held-out fold was Harrell’s concordance index (c-index). A final penalized Cox model was then fit on all complete cases using the CV-selected α to obtain linear-predictor risk scores. The risk score corresponds to the linear predictor of the elastic-net penalized Cox model. For patient *i*,

$$RiskScore_{i}=\sum\limits_{i=1}^{8}\beta_{j}X_{ij}$$

where ${X}_{ij}$denotes the standardized value of the *j*-th molecular feature and $\:{{\upbeta\:}}_{j}$​ are the coefficient estimates obtained from the final elastic-net Cox model fitted on the full TCGA-LIHC training dataset.

Elastic-net regularization was selected because the axis features (miRNA abundances and target-set activities) are not independent by construction, and correlated predictors are expected in this setting. In correlated feature spaces, pure L1 penalization can yield unstable selection, while pure L2 retains all predictors without sparsity. Elastic net provides a compromise between shrinkage and selection; the mixing parameter (α) was tuned within cross-validation [27].

### ML external validation

For external validation in GSE31384 [28], TCGA means and standard deviations were used to z-standardize the four miRNA abundances and the fixed TCGA coefficients were applied to compute a locked linear predictor. Discrimination was quantified by c-index from a Cox model with the continuous risk score and a 5-fold CV cutpoint was derived within GSE31384.

### Survival screening of union targets

Overall survival (OS) of 25 targets from the miRNA/mRNA pairs was analyzed using Kaplan–Meier curves with log-rank testing; hazard ratios (HRs) and 95% CIs were obtained from a univariable Cox model. Benjamini–Hochberg FDR adjustment was applied to log-rank *p*-values; significance threshold FDR ≤ 0.01. Plots display up to 120 months of follow-up with numbers at risk [29].

### Immune infiltration

Bulk expression matrices for TCGA-LIHC and GSE14520 were deconvolved to immune-cell fractions with CIBERSORT [30] and xCell (LM22, 22 subsets) and were visualized in R. For each immune subset and cohort, group differences were tested with Wilcoxon rank-sum tests; Benjamini–Hochberg FDR was applied across the 22 subsets. Effect size was defined as Δmean = mean(High) − mean(Low).

### Gene outcome analysis

The seven survival-prioritized genes were analyzed by TIMER2.0’s Gene Outcome module for LIHC [31]. Overall survival was modeled with a Cox proportional-hazards regression using gene expression as a continuous covariate. TIMER2.0 reports the Wald Z-score (sign indicates risk direction), hazard ratio and *P* value.

### Expression, methylation and stage profiling of *Uridine–Cytidine Kinase 2* (UCK2)

For *UCK2*, tumor–normal mRNA expression (log2 RSEM), DNA methylation (beta values) and pathologic stage comparisons were obtained from GSCA (https://guolab.wchscu.cn/GSCA/#/ ) using TCGA-LIHC data and default settings [14]. Group differences were taken from the resource’s built-in tests with Benjamini–Hochberg FDR control where provided. Reported statistics and P/FDR values were used directly.

### Hallmark gene-set for *UCK2*

Pathway context was assessed in GENI (https://www.genei.io/ ) by ranking all genes according to their correlation with *UCK2* across LIHC samples and running GSEA against MSigDB Hallmark sets. Normalized enrichment scores (NES) and FDR q-values were extracted to summarize positively and negatively associated programs [15].

### TISIDB analyses

TISIDB analyses were performed for TCGA-LIHC to relate *UCK2* expression to tumor immunity and druggability [16]. Associations between *UCK2* and inferred lymphocyte abundances were assessed using the resource’s immune-cell panels with Spearman rank correlations and two-sided *P* values reported as provided. Correlations between *UCK2* and curated immunomodulatory genes (immunoinhibitors, immunostimulators and MHC genes) were also evaluated. Differences in *UCK2* expression across the Thorsson immune subtypes (C1–C6) were tested using the Kruskal–Wallis procedure. The drug–gene network was queried to list compounds linked to *UCK2* and adjacent enzymatic targets.

### Single-cell transcriptomics and intercellular communication

HCC single-cell RNA-seq dataset GSE125449 [32] was processed in Seurat (R) [33]. Low-quality cells were removed using standard filters; counts were log-normalized, variable features were identified and dimensionality reduction used PCA (Principle Component Analysis) followed by UMAP. Cells were clustered with a shared-nearest-neighbor graph and annotated into major liver/tumor lineages using canonical markers.

### Cell-cell communication analysis

Cell–cell communication was inferred with CellChat R package (v1.6.1) [34]. Over-expressed ligands and receptors were identified using the Wilcoxon rank-sum test implemented within CellChat, with Bonferroni-adjusted *p* < 0.05. Communication probabilities between sender and receiver cell populations were computed using the default mass-action model. Statistical significance of inferred interactions was assessed by permutation testing (1000 permutations), and only interactions with adjusted *p* < 0.05 were retained for downstream analysis. Default normalization procedures accounting for cluster size were applied.

### Cell lines and culture conditions

HCC cell lines HepG2, Huh7, Hep3B2.1-7, and SNU-449 were obtained from the American Type Culture Collection (ATCC, Manassas, VA, USA). Cells were authenticated and maintained under standard conditions (37 °C, 5% CO₂) in Dulbecco’s Modified Eagle Medium (DMEM; Gibco, Thermo Fisher Scientific) supplemented with 10% fetal bovine serum (FBS; Gibco, Thermo Fisher Scientific) and 1% penicillin–streptomycin (Thermo Fisher Scientific).

### siRNA-mediated knockdown of *UCK2*

Cells were transfected with Silencer™ Select siRNAs targeting *UCK2* (Thermo Fisher Scientific) using Lipofectamine™ RNAiMAX Transfection Reagent (Thermo Fisher Scientific, Cat. #13778150) following the manufacturer’s protocol. Silencer™ Select Negative Control siRNA (Thermo Fisher Scientific, Cat. #4390846) was used as a control.

### Stable overexpression of *UCK2*

Gain-of-function studies were conducted by generating stable *UCK2*-overexpressing HCC cell lines. HepG2 and Huh7 cells were transduced with a lentiviral CMV-*UCK2* overexpression construct (OE-UCK2) packaged using a second-generation lentiviral system. Briefly, lentiviral particles were produced by co-transfecting HEK293T cells with the pLV-CMV-UCK2-Puro plasmid, psPAX2 packaging plasmid, and pMD2.G envelope plasmid (Addgene). Supernatants containing viral particles were collected at 48 and 72 h, filtered (0.45 μm), and used to infect target cells in the presence of 8 µg/mL polybrene (Sigma-Aldrich). Following transduction, cells were selected with puromycin (1–2 µg/mL) for 5–7 days to establish stable isogenic lines.

### RNA extraction and RT–qPCR

Total RNA was extracted 48 h post-transfection using TRIzol™ Reagent (Invitrogen, Cat. #15596026). Reverse transcription was performed with the High-Capacity cDNA Reverse Transcription Kit (Applied Biosystems, Cat. #4368814). RT–qPCR was carried out using TaqMan™ Universal Master Mix II, no UNG (Applied Biosystems, Cat. #4440040). The following TaqMan™ Gene Expression Assays were used: *UCK2* (Assay ID: Hs00989900_m1) and *GAPDH* (Assay ID: Hs99999905_m1) as an endogenous control. Reactions were run on an Applied Biosystems QuantStudio system, and relative quantification was calculated using the ΔΔCt method.

### Protein extraction and western blotting

Cells were lysed in RIPA Lysis and Extraction Buffer (Thermo Scientific, Cat. #89900), and protein concentrations were determined using the Pierce™ BCA Protein Assay Kit (Thermo Scientific, Cat. #23227). Equal amounts of protein were separated on NuPAGE™ 4–12% Bis-Tris gels (Invitrogen, Cat. #NP0321BOX) in MOPS SDS Running Buffer (Invitrogen, Cat. #NP0001) and transferred to PVDF membranes using iBlot™ 2 Transfer Stacks (Invitrogen, Cat. #IB24001). Membranes were blocked with SuperBlock™ (PBS) Blocking Buffer (Thermo Scientific, Cat. #37515) and incubated with primary antibodies against *UCK2* (Invitrogen, Cat. #PA5-14010) and *GAPDH* (Invitrogen, Cat. #MA5-15738). After washing, membranes were probed with HRP-conjugated secondary antibodies, goat anti-rabbit IgG (Thermo Scientific, Cat. #31460) or goat anti-mouse IgG (Thermo Scientific, Cat. #31430), and visualized using SuperSignal™ West Pico PLUS Chemiluminescent Substrate (Thermo Scientific, Cat. #34580).

### Cell proliferation assay

Proliferation was evaluated 24–72 h after transfection using the Cell Counting Kit-8 (CCK-8; Dojindo, Cat. #CK04). CCK-8 solution was added directly to each well, and plates were incubated for 1–4 h at 37 °C. Absorbance was measured at 450 nm with a microplate reader, and values were normalized to control groups.

### Colony formation assay

For clonogenic survival, 500–1,000 transfected cells were seeded into 6-well plates and cultured for 10–14 days. Colonies were fixed with methanol and stained with Crystal Violet (Thermo Fisher Scientific). Colonies containing > 50 cells were counted manually and compared with controls.

### Wound healing assay

At 48 h post-transfection, confluent monolayers in 6-well plates were scratched with a sterile 200 µL pipette tip to create a wound. Detached cells were removed by washing with PBS, and cultures were maintained in serum-reduced medium. Images were captured at 0 h and 24 h using a phase-contrast microscope. Wound closure was quantified by measuring the relative reduction in wound area over time compared with controls.

### Statistical analysis

All experiments were repeated at least three times, and results are shown as mean ± SD. Statistical tests were performed using GraphPad Prism 9. Two-tailed Student’s *t*-tests were used for two-group comparisons, and one-way or two-way ANOVA with appropriate post hoc tests was applied for multiple comparisons. P* < 0.05, P** < 0.01, P*** < 0.001 were considered statistically significant.

## Results

### Differential expression across cohorts

**Fig. 1:**
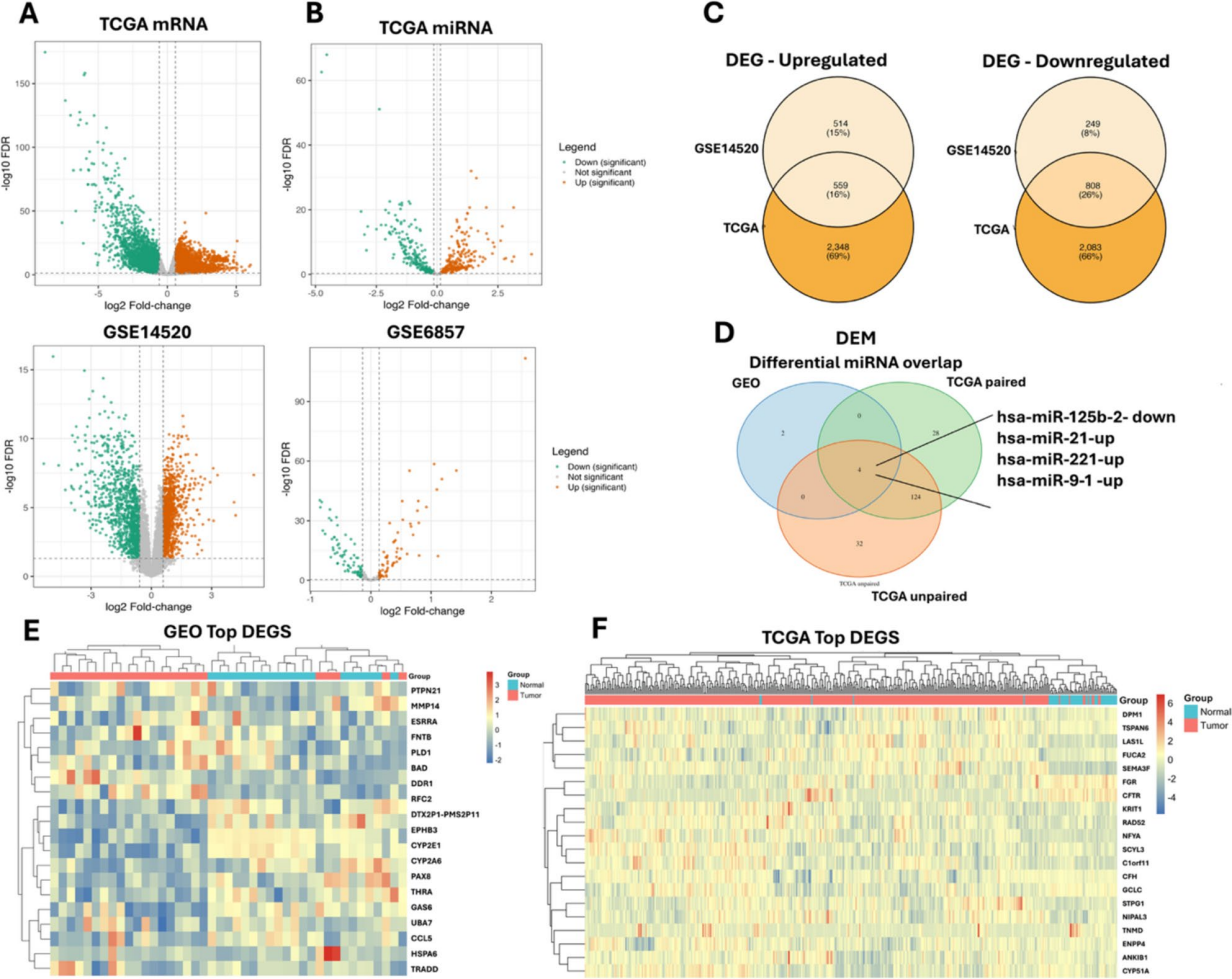
Transcriptome- and miRNome-level differential expression and cross-cohort concordance in HCC. **A** Differential gene expression between tumors and non-tumors in TCGA-LIHC RNA-seq (371 tumors, 50 normals) and GSE14520 microarray (24 tumors, 19 normals). **B** Differential miRNA expression in TCGA-LIHC miRNA-seq (372 tumors, 50 normals) and GSE6857 microarray (241 tumors, 241 normals). **C** Direction-specific Venn diagrams for genes. Overlap between TCGA and GSE14520 identifies 559 concordant Up and 808 concordant (**D)** miRNA overlap among TCGA (paired and unpaired models) and GSE6857 results in 4 overlapping miRNAs. **E–F** Row-scaled heatmaps of the top 20 DEGs per cohort show segregation of tumors from normals

At the mRNA level, TCGA-LIHC and GSE14520 showed widespread tumor–normal differences under FDR control (Fig. 1A) with 559 concordant up-regulated and 808 concordant down-regulated genes across cohorts (Fig. 1C). At the miRNA level, TCGA and GSE6857 likewise yielded multiple DEMs (Fig. 1B). Using direction-defined sets (≥ 1.5-fold), TCGA contributed 147 up / 141 down miRNAs and GSE6857 16 up / 15 down. Comparing TCGA (paired and unpaired) with GSE6857 highlighted a consistent overlapping set of four miRNAs i.e., miR-125b-2 (downregulated), miR-21 (upregulated), miR-221 (upregulated), and miR-9-1 (upregulated) (Fig. 1D). Row-scaled heatmaps of the top 20 DEGs per cohort showed clear tumor–normal separation (Fig. 1E–F).

### miRNA–mRNA network and functional enrichment

**Fig. 2:**
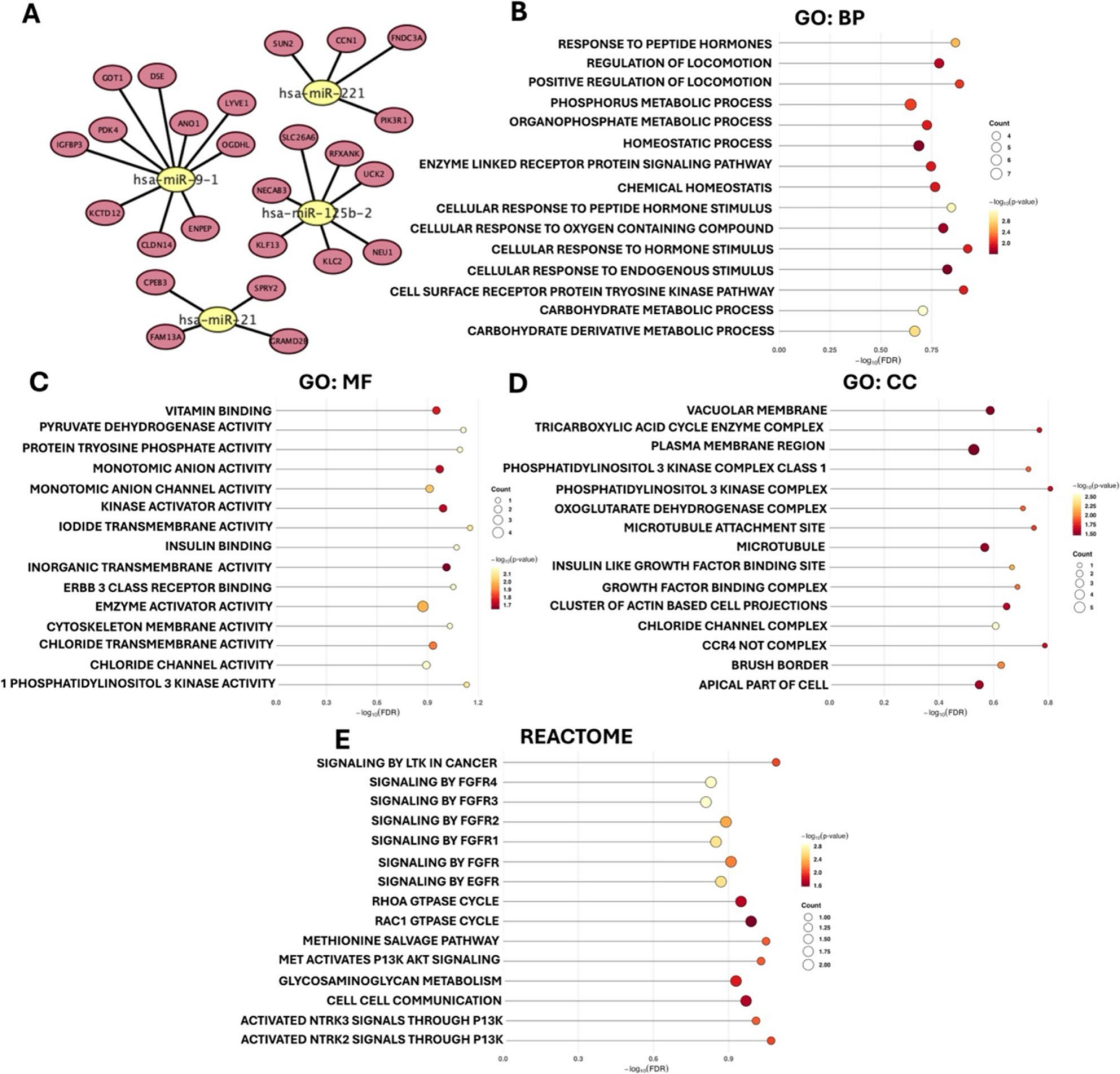
miRNA–mRNA network and enrichment of DEG-supported targets. **A** Bipartite network of 25 DEG-supported regulatory pairs derived from TargetScan ∩ MiRDB ∩ meta-DEG. **B** GO: Biological Process: hormone/insulin responses and carbohydrate/metabolic programs. **C** GO: Molecular Function: ion channel/transport and kinase-regulatory activities with receptor binding. **D** GO: Cellular Component: plasma/vacuolar membranes, class I PI3K complexes, microtubule apparatus, IGF-binding complex. **E** Reactome: RTK (FGFR/EGFR/MET) signaling to PI3K–AKT, RHO/RAC cycles, cell–cell communication and glycosaminoglycan metabolism

The bipartite network (Fig. 2A) shows the 25 miRNA/mRNA pairs. GO-BP terms clustered around peptide-hormone/insulin responses and carbohydrate/phosphorus metabolism pointing to endocrine and metabolic rewiring (Fig. 2B). GO-MF was enriched for ion transport/channel activities (chloride/iodide), kinase-activator and protein-tyrosine-phosphatase-activator activities and receptor binding (Fig. 2C). GO-CC emphasized plasma/vacuolar membranes, class I PI3K complexes, microtubule structures and the insulin-like growth factor binding complex (Fig. 2D). Reactome converged on RTK→PI3K/AKT pathways (FGFR/EGFR/MET modules), RHO/RAC GTPase cycles, cell–cell communication and glycosaminoglycan metabolism (Fig. 2E). No category crossed FDR < 0.05 given the small gene set; therefore, these are reported as prioritized, biologically consistent trends that nominate growth-factor/RTK signaling, membrane transport and carbohydrate/energy metabolism as the dominant axes in the network.

### Coherence and survival direction

**Fig. 3:**
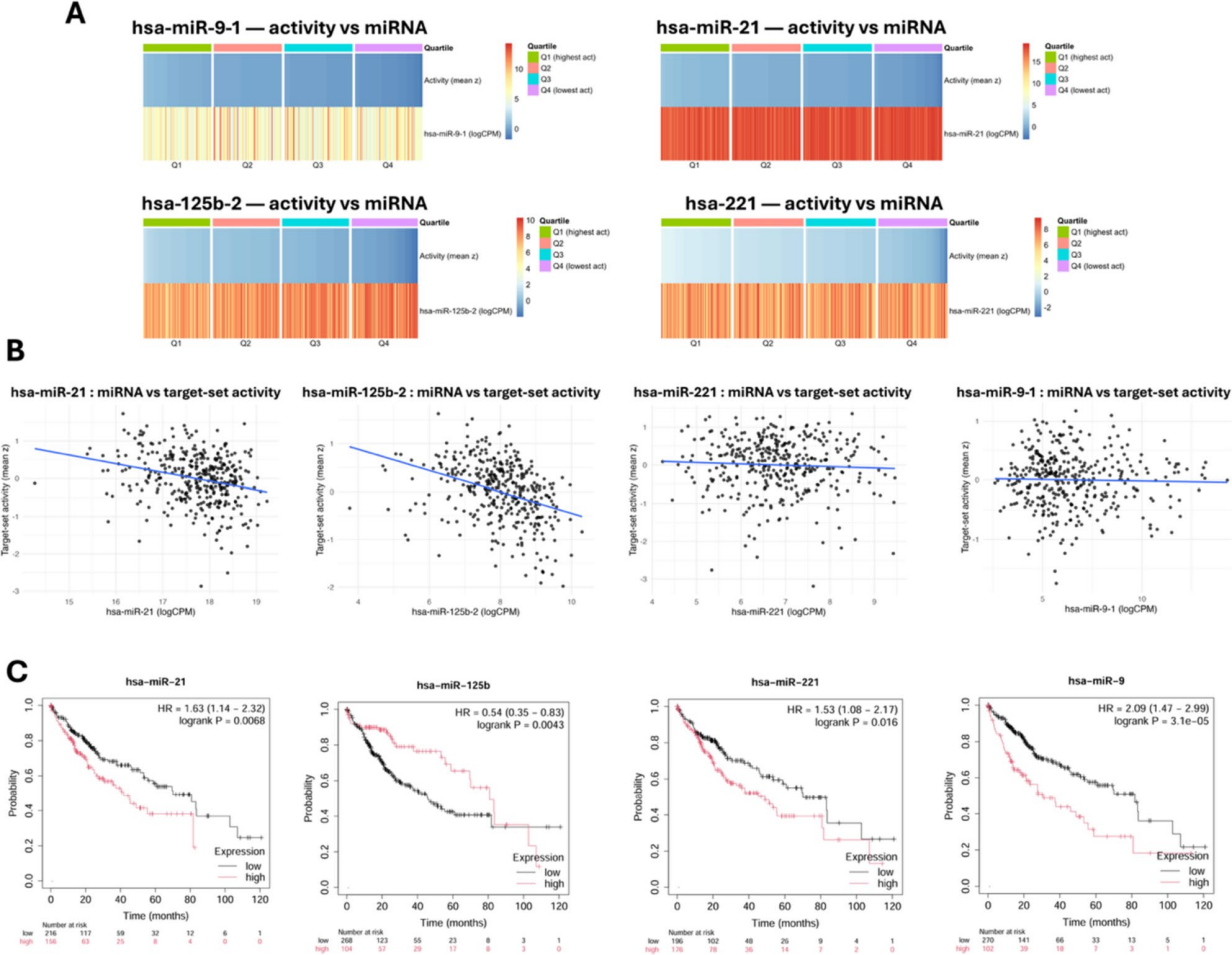
Activity–abundance coherence and survival direction of candidate miRNAs.** A** Two-track heatmaps per miRNA in TCGA tumors; columns ordered by decreasing target-set activity with quartiles Q1–Q4. Q1–Q4 represent quadrants defined by median-based dichotomization of miR-125b-2 and UCK2 expression levels: Q1 (miR-125b-2^High/UCK2^High), Q2 (miR-125b-2^Low/UCK2^High), Q3 (miR-125b-2^Low/UCK2^Low) and Q4 (miR-125b-2^High/UCK2^Low). **B** Scatterplots showing Spearman coherence between miRNA abundance (logCPM) and target-set activity (mean z), with least-squares trend lines. **C** KM-plotter overall-survival curves for family-level miRNAs using the hazard ratio and log-rank *P*

miR-125b-2 showed the strongest inverse association with its target-set activity (*ρ* = −0.41; *P* < 1 × 10⁻¹²), with activity decreasing from Q1 to Q4 while abundance increased across the same ordering; the scatter plot displayed a steep negative slope. miR-21 also showed negative coherence of smaller magnitude (*ρ* = −0.26; *P* = 7.1 × 10⁻⁷). miR-221 and miR-9-1 were near-null under the current supported target sets (miR-221: *ρ* = −0.04, *P* = 0.44; miR-9-1: *ρ* = −0.03, *P* = 0.51). After Benjamini–Hochberg adjustment across the four correlations, miR-125b-2 and miR-21 remained significant, whereas miR-221 and miR-9-1 did not (Fig. 3A-B). In KM-plotter, higher expression of miR-21, miR-221, and miR-9 associated with worse overall survival (hazard ratios > 1; log-rank significant), whereas higher miR-125b associated with better overall survival (hazard ratio < 1), consistent with the up/oncomiR versus down/tumor-suppressor directions in differential expression (Fig. 3C). Overall, the strongest cohort-level inverse signal was observed for the miR-125b-2 axis, with miR-21 providing secondary support; miR-221 and miR-9-1 did not show cohort-level coherence in this analysis.

### Machine Learning (ML) prognostic miRNA axes in LIHC

**Fig. 4:**
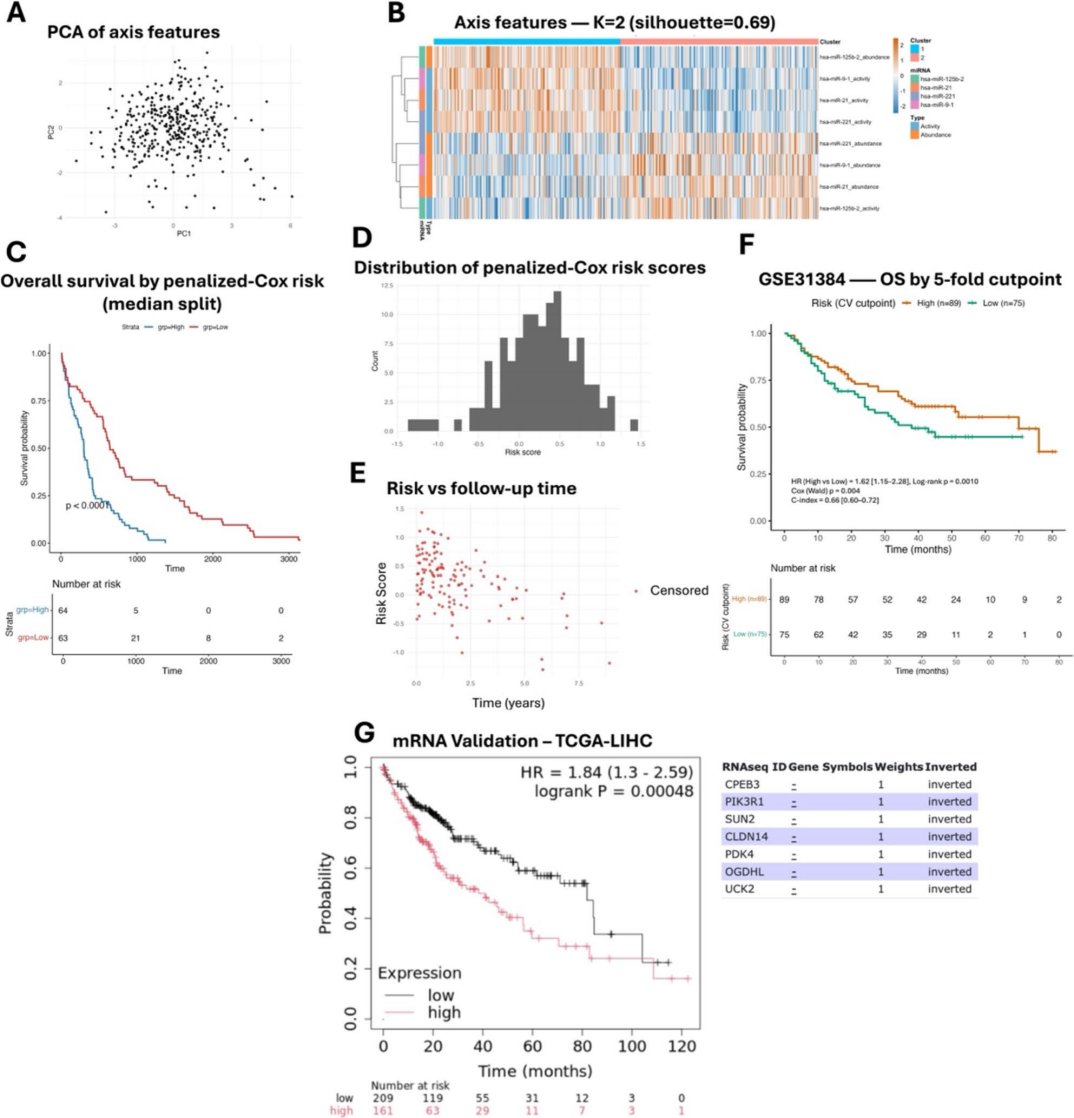
Machine-learning analysis of axes features and external cohort. **A** PCA of the 8 axis features (abundance + activity). **B **Heatmap of the 8 features ordered by k-means K = 2; annotations indicate miRNA and feature type. **C** TCGA OS by median split of the final penalized-Cox risk score. **D** Distribution of fitted risk scores (all 127 cases). **E** Risk vs. follow-up time with censoring marks. **F** GSE31384 OS using a 5-fold CV cutpoint (**G**) Validation of the mRNA component in TCGA-LIHC independently

PCA of the eight axis features shows a broad, diffuse cloud (Fig. 4A). k-means clustering with K = 2 yields a moderate partition (silhouette = 0.69); the heatmap displays graded, alternating patterns (Fig. 4B). In TCGA-LIHC, a Kaplan–Meier curve based on the median of the penalized-Cox risk score shows clear separation of overall survival with log-rank *p* < 0.0001 (Fig. 4C). The fitted risk scores are approximately unimodal (Fig. 4D) and tend to be higher among earlier events in the risk–follow-up scatter (Fig. 4E). In the external cohort GSE31384, a 5-fold cross-validated cutpoint defines High (*n* = 89) and Low (*n* = 75) groups with significant separation (HR = 1.62 [1.15–2.28], log-rank *p* = 0.0010; Cox [Wald] *p* = 0.004), and c-index = 0.66 [0.60–0.72] (Fig. 4F). Furthermore, validation of the mRNA component in TCGA-LIHC independently demonstrated significant survival discrimination (HR = 1.84, 95% CI: 1.30–2.59, log-rank *p* = 0.00048), supporting the contribution of the downstream transcriptomic axis (Fig. 4G). Overall, the axis features do not define discrete molecular subtypes, but a composite risk score does stratify survival in TCGA and shows a significant High/Low split in the external cohort when using a cross-validated cutpoint.

### Survival-prioritized targets and coherence with miRNA direction

**Fig. 5:**
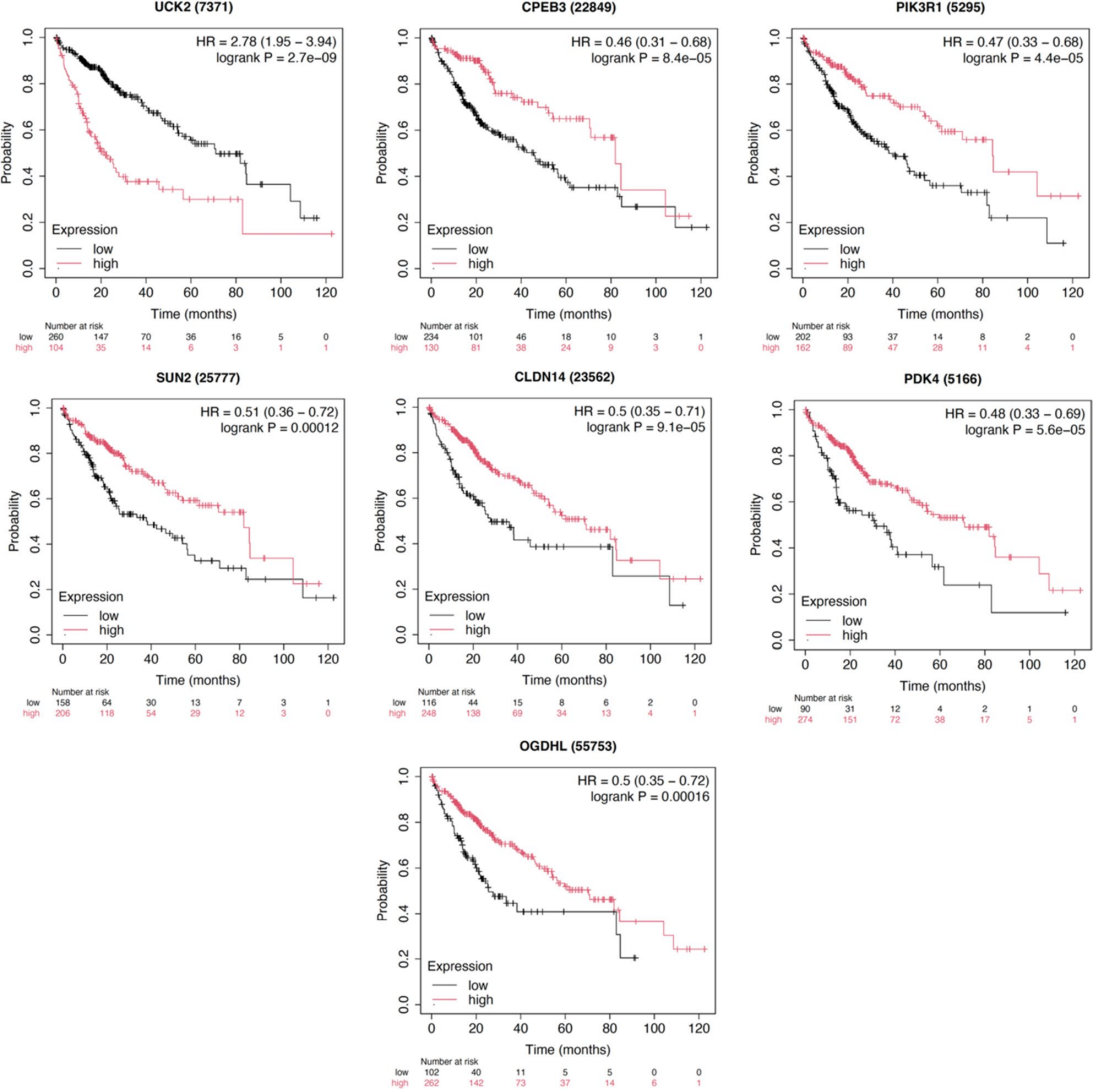
Kaplan–Meier survival of shortlisted union targets. Panels show OS for seven genes that passed FDR ≤ 1% after testing 25 targets. *UCK2* (target of miR-125b-2) displays adverse association with high expression (risk). CPEB3 (miR-21 target), PIK3R1 and SUN2 (miR-221 targets), and CLDN14, PDK4, OGDHL (miR-9-1 targets) display protective associations with high expression

Seven of 25 genes met FDR ≤ 1% and were shortlisted (Fig. 5). *UCK2* high expression associated with worse OS (HR = 2.78, 95% CI 1.95–3.94; log-rank *P* = 2.7 × 10⁻⁹). Six genes showed protective associations when highly expressed: CPEB3 (HR = 0.46, 0.31–0.68; *P* = 8.4 × 10⁻⁵), PIK3R1 (0.47, 0.33–0.68; *P* = 4.4 × 10⁻⁵), SUN2 (0.51, 0.36–0.72; *P* = 1.2 × 10⁻⁴), CLDN14 (0.50, 0.35–0.71; *P* = 9.1 × 10⁻⁵), PDK4 (0.48, 0.33–0.69; *P* = 5.6 × 10⁻⁵) and OGDHL (0.50, 0.35–0.72; *P* = 1.6 × 10⁻⁴). *UCK2* behaves as an oncogene (high→worse OS). The other six (CPEB3, PIK3R1, SUN2, CLDN14, PDK4, OGDHL) behave like Tumor-suppressive Genes (high→better OS), consistent with repression by upregulated oncomiRs (Fig. 5).

### Immune infiltration analysis

**Fig. 6:**
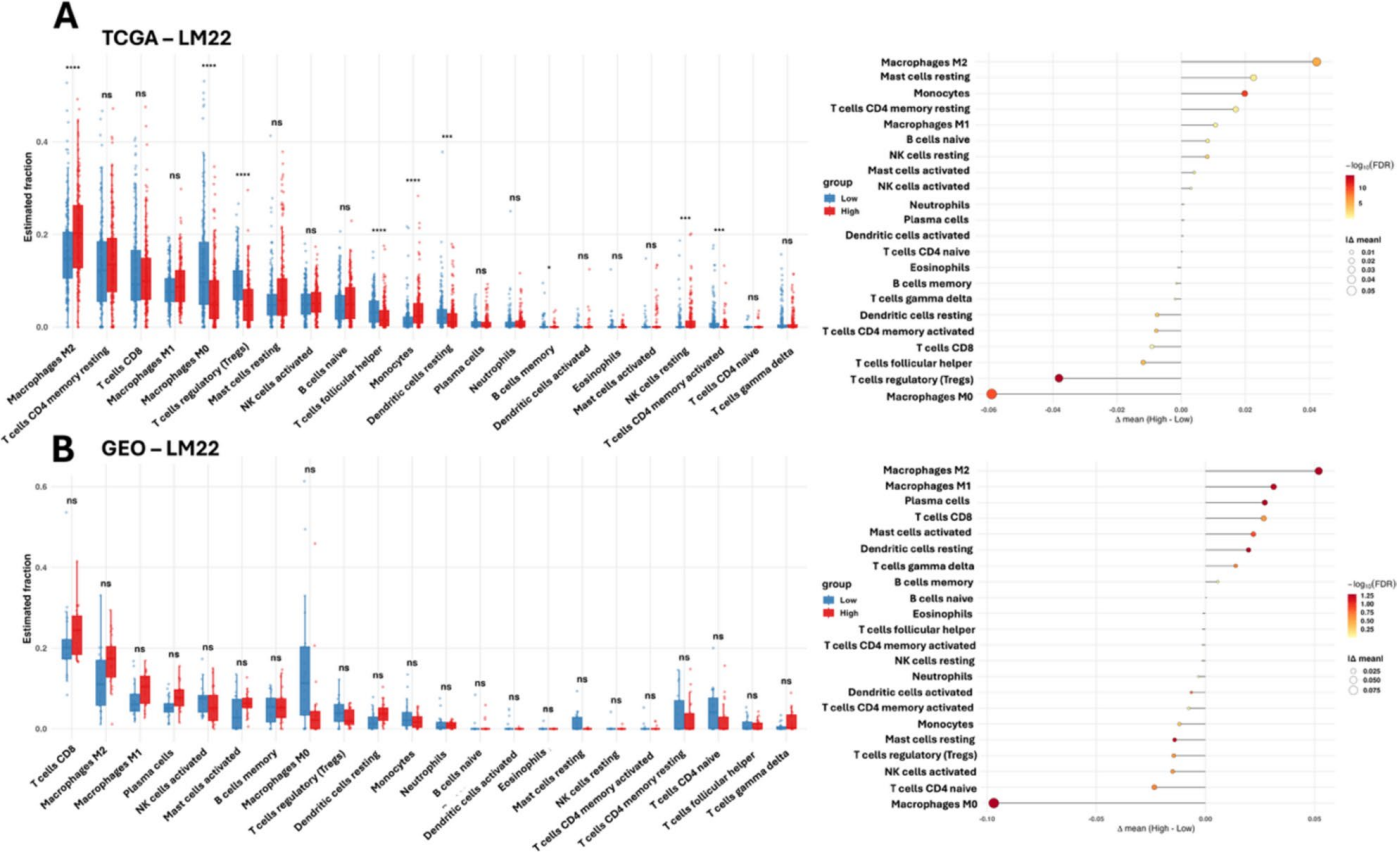
Immune infiltration (CIBERSORT–LM22) by gene-expression strata.** A** TCGA-LIHC. Boxplots (left) show estimated fractions by Low vs. High expression (pooled across the seven genes); asterisks mark FDR-adjusted significance. Macrophages M0 decrease in High, while M1/M2, B-cell/NK/mast-cell subsets tend to increase. **B** GSE14520. A consistent pattern is observed with stronger depletion of M0 and increases in M1/M2, CD8 T cells, plasma cells and activated mast cells

Across TCGA-LIHC, high strata showed lower Macrophages M0 and modest increases in M1/M2 macrophages, memory B cells, NK cells (resting/activated), mast cells (resting/activated) and monocytes (Δmean > 0; several subsets FDR-significant), whereas Tregs tended to be reduced (Δmean < 0) (Fig. 6A). In GSE14520, the pattern was directionally similar with even larger effects for Macrophages M0 (depleted in High) and enrichment of M1/M2 macrophages, CD8 T cells, plasma cells and activated mast cells (Fig. 6B). Together, both cohorts indicate a shift from undifferentiated M0 toward polarized M1/M2 macrophages and greater lymphoid activity in the High strata. This pattern is consistent with the protective targets (CPEB3, PIK3R1, SUN2, CLDN14, PDK4, OGDHL) aligning with a more activated/antitumor microenvironment when highly expressed.

**Fig. 7:**
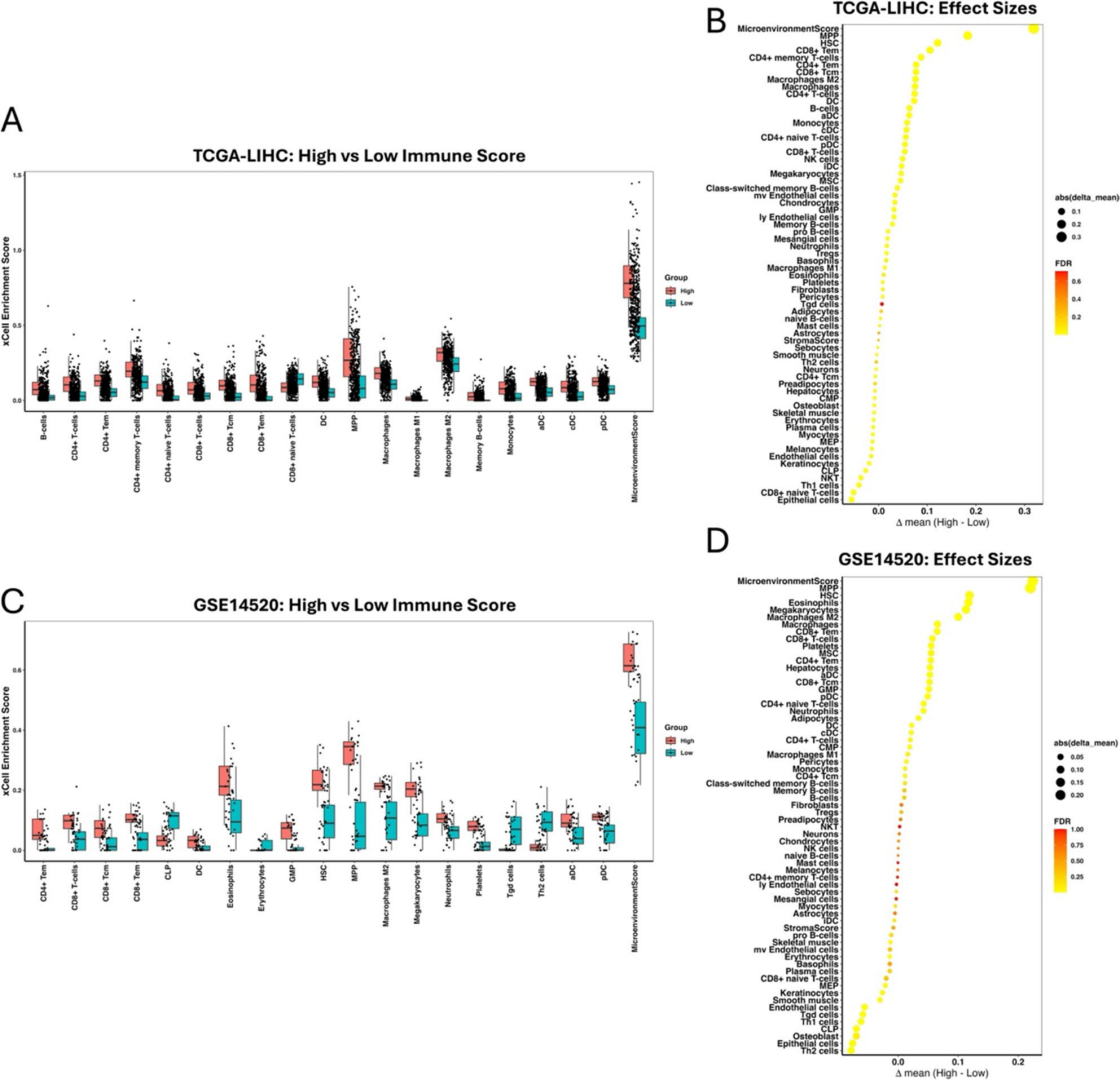
Immune infiltration (xCELL–LM22) by gene-expression strata. **A** TCGA-LIHC. Boxplots (left) show estimated fractions by Low vs. High expression (pooled across the seven genes); asterisks mark FDR-adjusted significance. **B** GSE14520. A consistent pattern is observed with stronger in box plot and dot plot

To validate the robustness of immune infiltration patterns identified using CIBERSORT, we performed independent immune deconvolution using xCell in both TCGA-LIHC and GSE14520 cohorts (Fig. 7A-B). Tumor samples exhibited significant remodeling of the immune microenvironment relative to control tissues. In TCGA-LIHC, macrophage-related signatures demonstrated elevated enrichment scores in tumors. Stromal and microenvironment-associated components were also enriched in tumor tissues. In the GSE14520, xCell recapitulated similar directional trends. Macrophage-associated signatures, plasma cells and activated T cell subsets exhibited tumor-associated enrichment, while selected naïve and resting immune populations showed relative reduction.

### Risk signal and characterization of *UCK2*

**Fig. 8:**
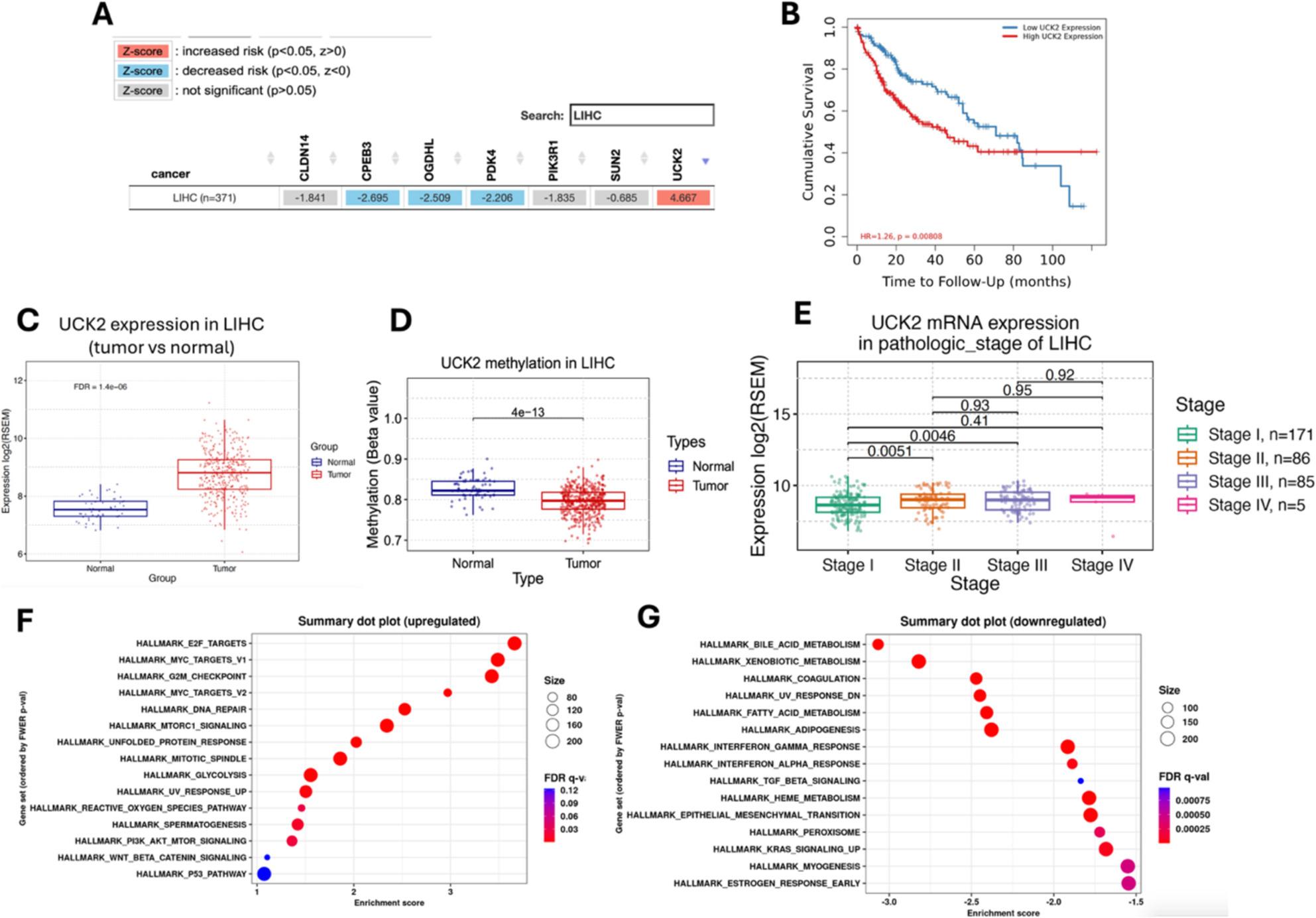
External characterization of survival-prioritized targets. **A **TIMER2.0 Gene Outcome Z-scores in LIHC: red indicates increased risk (positive Z), blue decreased risk (negative Z); *UCK2* shows the largest positive Z. **B **Kaplan–Meier overall survival for *UCK2* (high vs. low expression by TIMER2.0 default split), showing worse survival with high expression (HR = 1.26; log-rank *p* = 0.0081) **(C)** GSCA tumor vs. normal expression of *UCK2* (higher in tumors). **D** GSCA methylation of *UCK2* (lower in tumors). **(E)** GSCA pathologic-stage expression of *UCK2* (pairwise *P* values annotated). **F–G** GENI Hallmark enrichment for genes correlated with *UCK2*: positively associated proliferative programs (**F**) and negatively associated metabolic/immune programs (**G**)

Across the seven genes in TIMER2.0, *UCK2* showed the strongest increased-risk association (Z = + 4.667), whereas the remaining genes displayed decreased-risk signals with negative Z-scores (range approximately − 0.7 to − 2.7) (Fig. 8A). A representative Kaplan–Meier curve for *UCK2*: the high-expression group has worse overall survival than the low-expression group (HR = 1.26, log-rank *p* = 0.0081), consistent with the positive risk direction (Fig. 8B). In GSCA, *UCK2* expression was higher in tumors than in adjacent normal liver (log2 RSEM; FDR = 1.4 × 10⁻^5^) (Fig. 8C) and exhibited lower methylation in tumors (*P* = 4 × 10⁻¹³), indicating hypomethylation (Fig. 8D). Stage-wise analysis showed a step-up from stage I to stage II/III (pairwise *P* ≈ 0.005–0.005), with no clear differences among later stages (Fig. 8E).

GENI positioned *UCK2* within proliferative and metabolic programs: positively correlated Hallmarks included E2F targets, MYC targets (V1/V2), G2M checkpoint, mTORC1 signaling, unfolded-protein response, mitotic spindle and glycolysis (Fig. 8F), whereas negatively correlated Hallmarks included bile-acid/xenobiotic metabolism, coagulation, interferon responses, heme metabolism, epithelial–mesenchymal transition, peroxisome, KRAS signaling, myogenesis and early estrogen response (Fig. 8G).

### Immune correlates and drug links of *UCK2*

**Fig. 9:**
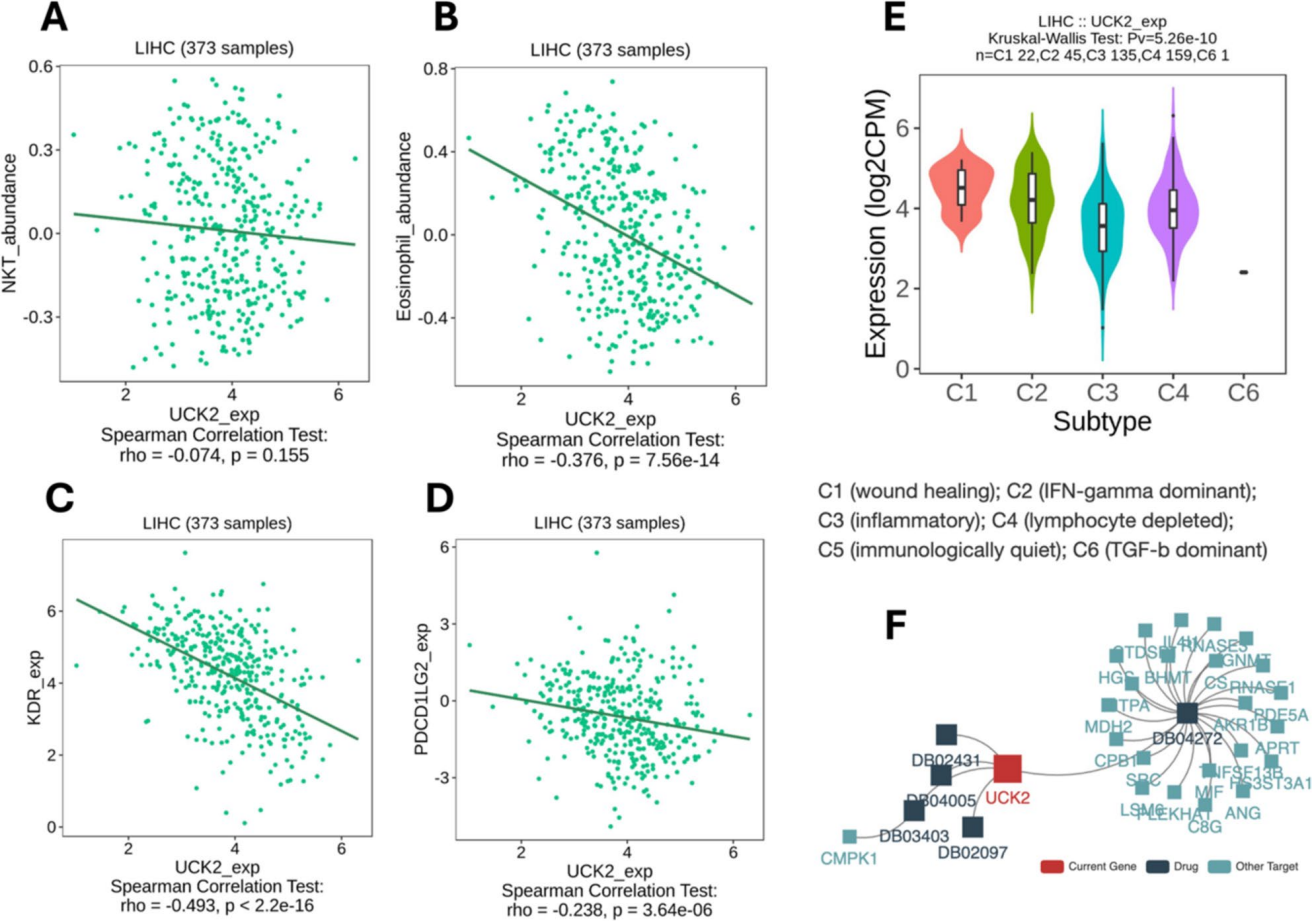
TISIDB analyses for *UCK2 *in LIHC.** A** Spearman correlations between *UCK2* expression and inferred lymphocyte abundances: significant negative association with eosinophils and NKT cells. **B** Correlations with immunomodulators: negative associations with KDR (VEGFR2) and PDCD1LG2 (PD-L2). **C ***UCK2* expression across immune subtypes (C1 wound-healing, C2 IFN-γ–dominant, C3 inflammatory, C4 lymphocyte-depleted, C5 immunologically quiet, C6 TGF-β–dominant); global Kruskal–Wallis *P* = 5.26 × 10⁻¹⁰; expression highest in C4. **D** DrugBank-derived network showing *UCK2*-linked small molecules and adjacent targets (red = *UCK2*, teal = other targets, squares = drugs)

Among lymphocyte subsets, eosinophil abundance and NKT cells showed the highest significant negative correlation with *UCK2* (eosinophil: *ρ* = −0.376, *P* = 7.56 × 10⁻¹⁴, NKT: *ρ* = −0.074, *P* = 0.155) (Fig. 9A). For immunomodulators, *UCK2* correlated negatively with KDR/VEGFR2 (*ρ* = −0.493, *P* < 2.2 × 10⁻¹⁶) and with PDCD1LG2 (PD-L2) (*ρ* = −0.238, *P* = 3.64 × 10⁻⁶) (Fig. 9B). *UCK2* expression varied across immune subtypes with a highly significant global difference (Kruskal–Wallis *P* = 5.26 × 10⁻¹⁰): expression was highest in C4 (lymphocyte-depleted) and lower in C1/C2/C3; C6 contained only one sample and is not interpretable (Fig. 9C). The drug–gene network connected *UCK2* to several DrugBank compounds (e.g., DB02097, DB03403, DB04005, DB02431) and to neighboring nucleotide-metabolism proteins (e.g., CMPK1) indicating chemically tractable space around *UCK2* but not constituting efficacy evidence (Fig. 9D).

### Epithelial *UCK2* linked to ECM/GF signaling

**Fig. 10:**
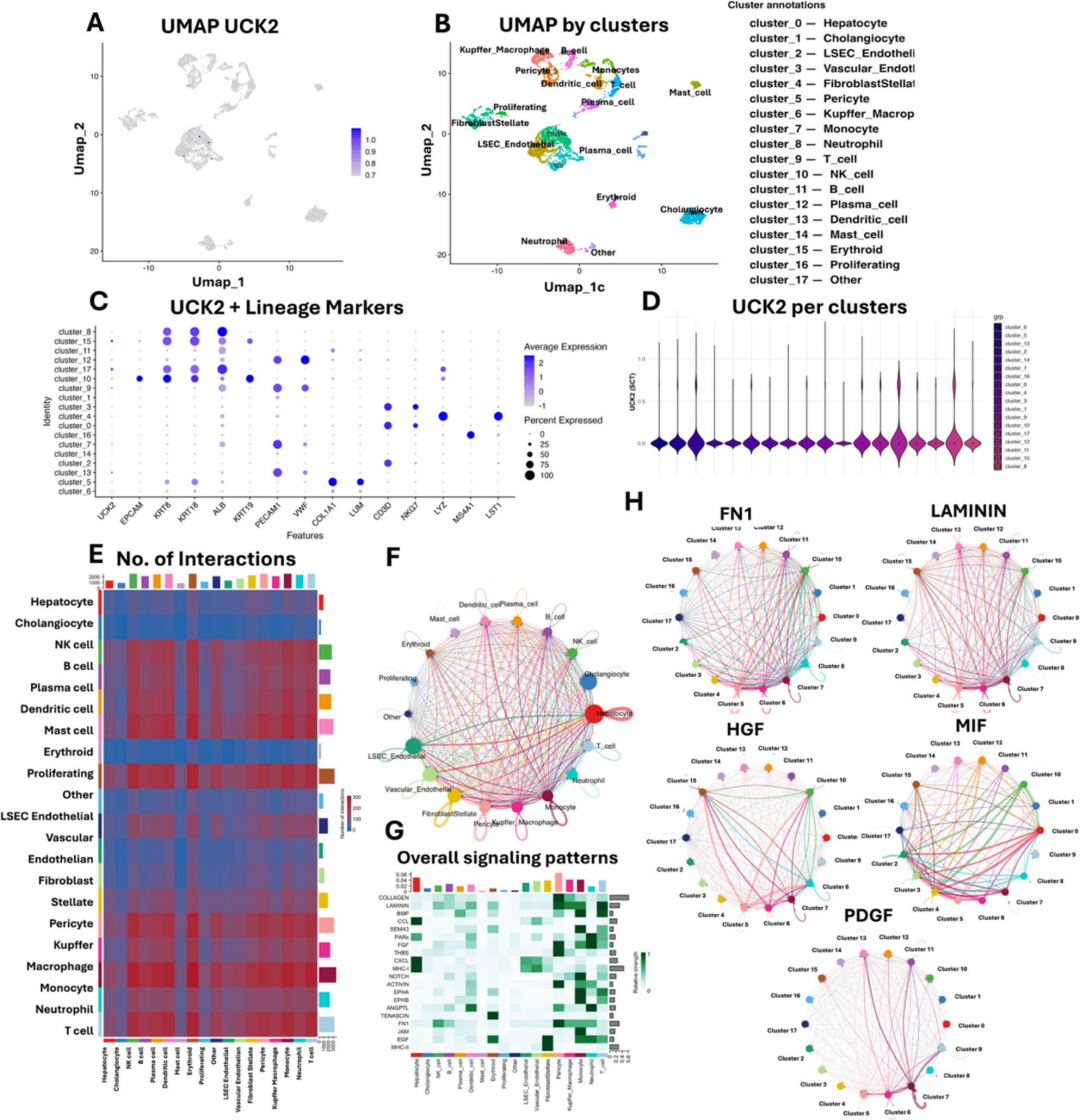
Single-cell distribution of *UCK2 *and CellChat networks in HCC. **A** UMAP feature plot of *UCK2*. **B** UMAP colored by annotated clusters (hepatocyte-like, cholangiocyte, endothelial/vascular, fibroblast/stellate, pericyte, Kupffer/macrophage, monocyte, neutrophil, T, NK, B, plasma, dendritic, mast, erythroid, proliferating, other). **C** Dot plot of *UCK2* with lineage markers (dot size = % cells; color = average expression). **D** Violin plots of *UCK2* across clusters. **E** Heatmap of the number of inferred interactions between lineages. **F** Global CellChat circle network (edge width ∝ interaction strength). **G** Heatmap of overall signaling patterns aggregated to pathways. **H** Pathway-specific circle plots for FN1, LAMININ, HGF, MIF and PDGF signaling

UMAP feature plotting showed *UCK2* expression concentrated within epithelial compartments, with signal in hepatocyte-like malignant clusters and cholangiocytes and low to negligible in immune or stromal lineages (Fig. 10A–B). The dot plot combining *UCK2* with canonical lineage markers confirmed co-occurrence with epithelial markers and absence in hematopoietic and endothelial signatures (Fig. 10C). Violin plots by cluster highlighted higher *UCK2* in hepatocyte-like and cholangiocyte clusters and minimal expression in macrophages, lymphocytes and stromal populations (Fig. 10D).

The interaction heatmap indicated dense communication between epithelial, endothelial/vascular and fibroblast/stellate compartments, with additional links to myeloid and lymphoid cells (Fig. 10E). The global circle network showed epithelial clusters as prominent senders and receivers (Fig. 10F). Pathway summarization pointed to extracellular-matrix and growth-factor programs as major conduits (Fig. 10G). In pathway-specific networks, strong traffic was observed through FN1 (fibronectin) and LAMININ signaling, as well as HGF, MIF, and PDGF pathways (Fig. 10H) showing stromal–epithelial crosstalk and matrix remodeling alongside mitogenic signaling.

### *UCK2* regulates proliferation, clonogenicity, and migration in HCC via bidirectional genetic manipulation

**Fig. 11:**
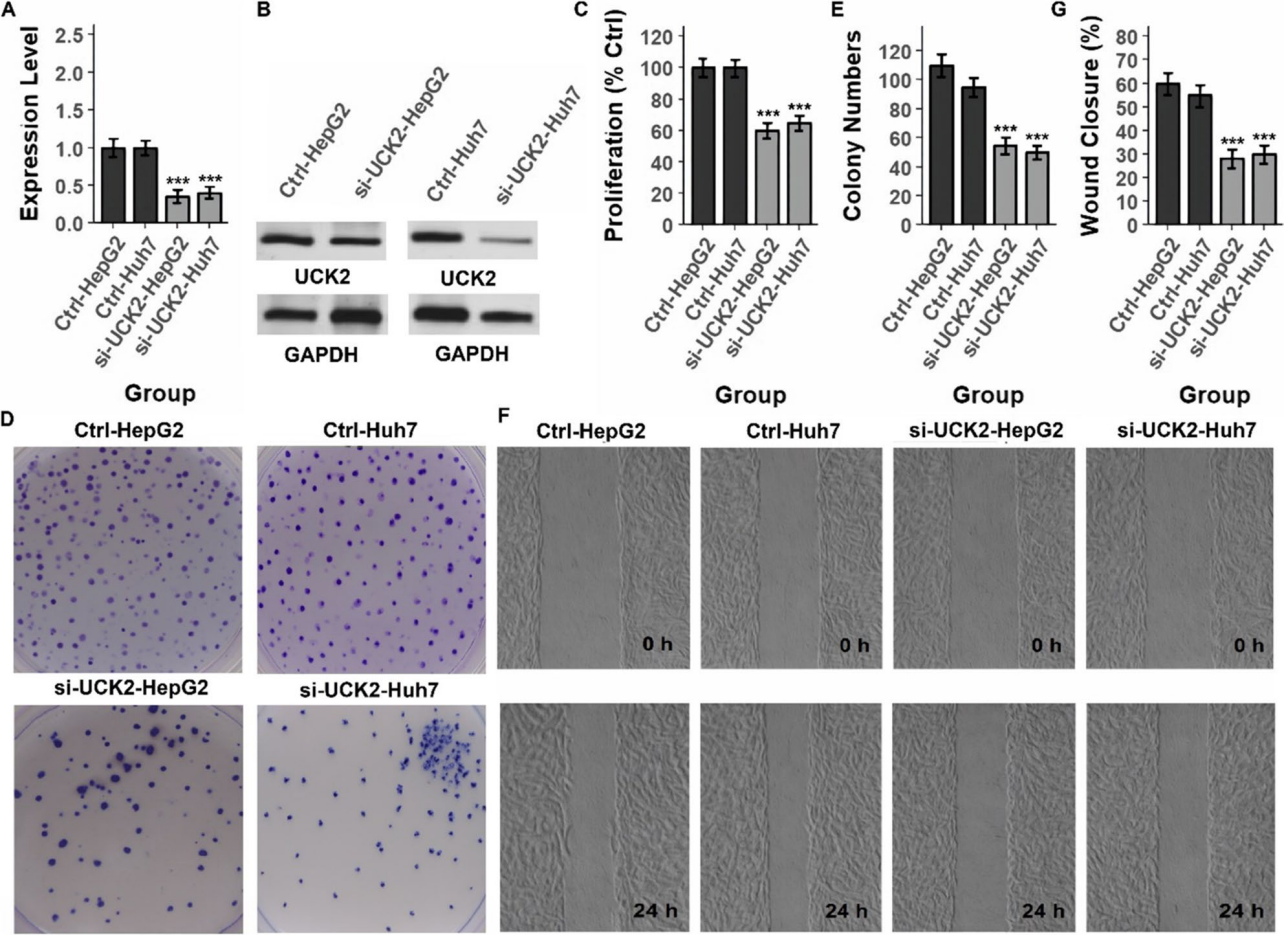
*UCK2 *knockdown suppresses malignant phenotypes in HepG2 and Huh7 cells. **A** RT-PCR analysis showing significant reduction of UCK2 mRNA levels following siRNA-mediated knockdown in HepG2 and Huh7 cells compared with control groups. **B** Western blot analysis confirming decreased *UCK2* protein expression upon siRNA treatment. **C** Proliferation assays (CCK-8) showing reduced cell proliferation in si-*UCK2* groups relative to controls. **D**, **E** Representative images and quantification of colony formation assays, demonstrating markedly decreased clonogenic capacity after *UCK2* knockdown. **F**, **G** Representative wound healing images at 0 h and 24 h and quantitative analysis of wound closure rates, revealing impaired migration following *UCK2* silencing. Data are presented as mean ± SD, ***P < 0.001 vs. control

**Fig. 12:**
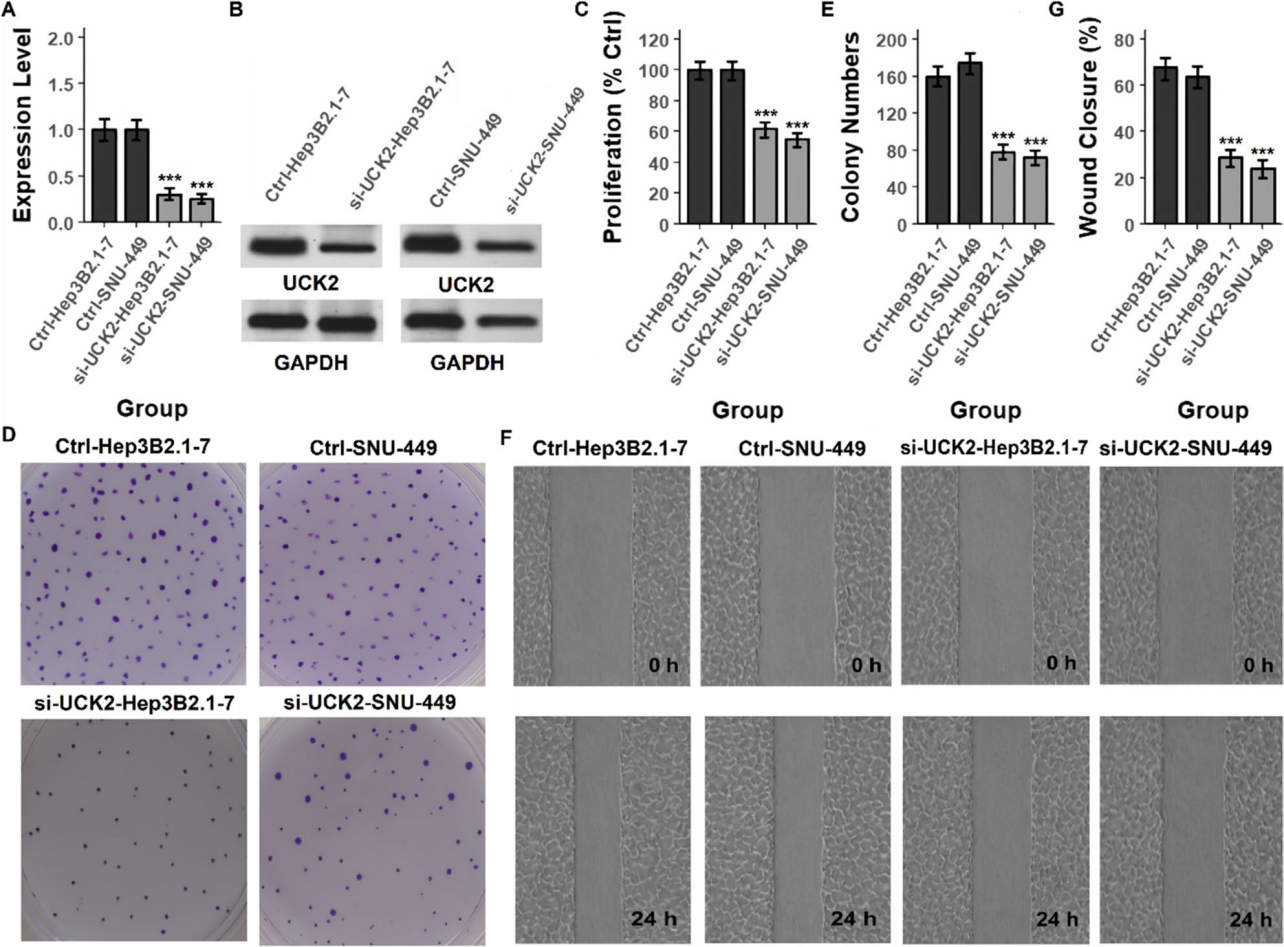
*UCK2 *knockdown reduces proliferation, clonogenicity, and motility in Hep3B2.1-7 and SNU-449 cells.**A** RT-PCR analysis confirming significant downregulation of *UCK2* mRNA levels in siRNA-transfected Hep3B2.1-7 and SNU-449 cells. **B** Western blot validation showing reduced *UCK2* protein levels following knockdown. **C** Proliferation assays (CCK-8) indicating a significant decrease in cell growth after *UCK2* silencing. **D**, **E** Representative colony formation assays and quantification, showing suppression of clonogenic potential in *UCK2*-deficient cells compared with controls. **F**, **G** Wound healing assays at 0 h and 24 h and corresponding quantification, demonstrating impaired cell migration upon *UCK2* knockdown. Data are presented as mean ± SD, ***P < 0.001 vs. control

**Fig. 13:**
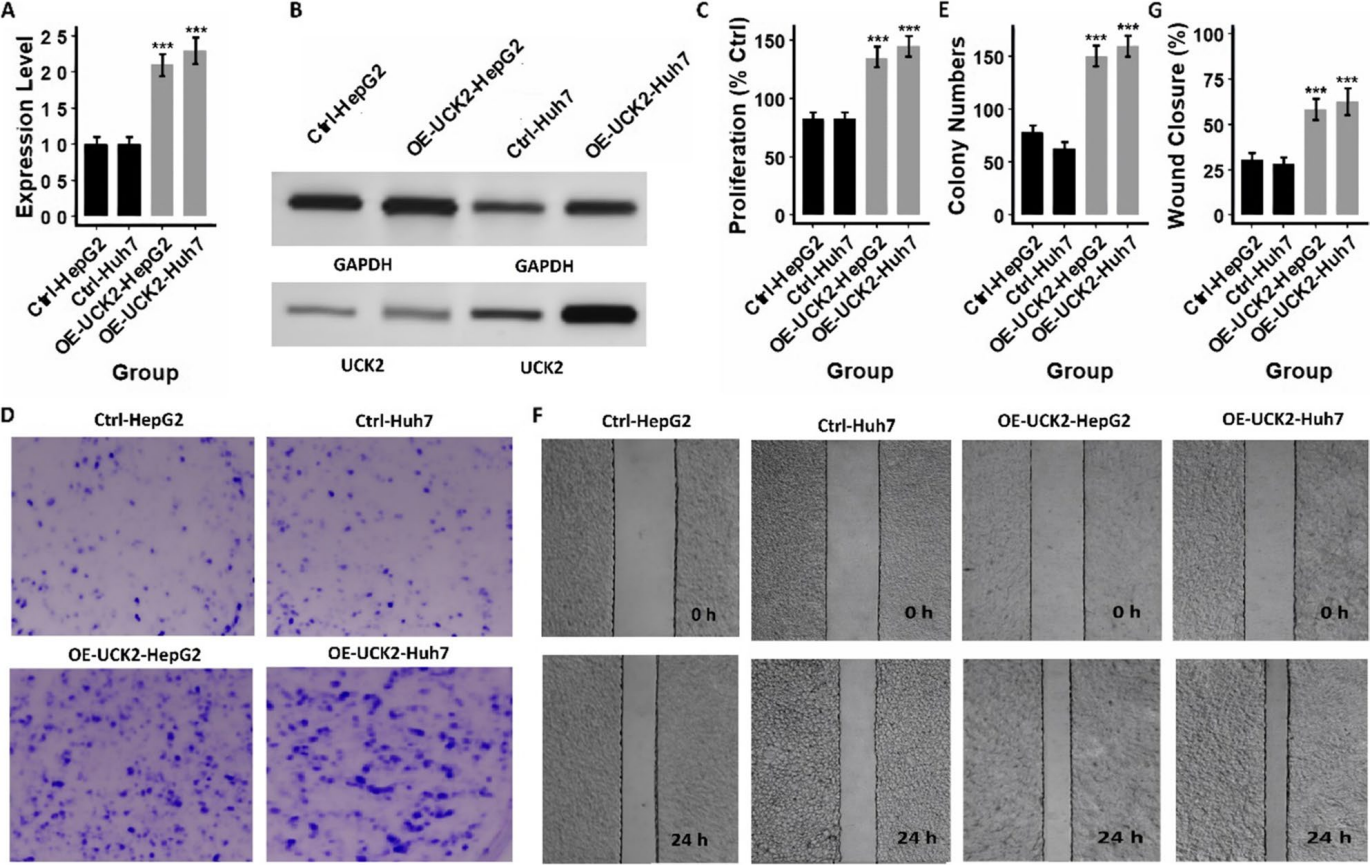
*UCK2* overexpression enhances proliferation, clonogenicity, and migration in HCC cells. **A** Quantitative RT–qPCR analysis confirming robust overexpression of *UCK2* in HepG2 and Huh7 cells following lentiviral transduction with the *UCK2* overexpression construct (OE-*UCK2*). **B** Representative Western blot images validating *UCK2* protein overexpression in OE-*UCK2* HepG2 and Huh7 cells. **C** Overexpression of *UCK2* significantly increases proliferation in both HepG2 and Huh7 cell lines. **D**–**E** Colony formation assay demonstrating that OE-*UCK2* HepG2 and Huh7 cells form markedly more colonies compared with their matched controls. **F**–**G** Wound healing assays showing accelerated migration in *UCK2*-overexpressing cells. Data are presented as mean ± SD, ***P < 0.001 vs. control

To functionally validate the biological role of *UCK2* in HCC, we first performed siRNA-mediated knockdown experiments in four HCC cell lines (HepG2, Huh7, Hep3B2.1-7, and SNU-449). As shown in Figs. 11A–B and 12A–B, Supplementary Fig. 1–2, siRNA treatment markedly reduced UCK2 mRNA and protein expression, confirming efficient gene silencing across all tested lines. *UCK2* knockdown significantly decreased cellular proliferation (Figs. 11C and 12C, and Supplementary Table), consistent with the transcriptomic evidence linking *UCK2* to proliferative Hallmark pathways. Clonogenic assays demonstrated that si-*UCK2* cells formed substantially fewer colonies compared with control groups (Figs. 11D–E and 12D–E, and Table S1), indicating that *UCK2 i*s required for maintaining long-term growth potential. Furthermore, wound-healing assays revealed impaired migratory capacity in *UCK2*-silenced cells, with markedly reduced gap closure at 24 h relative to controls (Figs. 11F–G and 12F–G, and Supplementary Table). Collectively, these loss-of-function data indicate that *UCK2* promotes cell proliferation, clonogenicity, and motility in HCC. To complement the knockdown studies, we next generated stable isogenic *UCK2*-overexpressing HCC models (HepG2 and Huh7) using a lentiviral *UCK2* expression construct. RT-qPCR and Western blot analyses confirmed robust *UCK2* overexpression compared with empty-vector controls (Fig. 13A–B and Supplementary Table). In direct contrast to the knockdown phenotype, *UCK2* overexpression significantly increased proliferation in both cell lines (Fig. 13C and Supplementary Table), further supporting a pro-growth role for *UCK2*. Clonogenic assays demonstrated a marked increase in colony number and density in *UCK2*-overexpressing HepG2 and Huh7 cells (Fig. 13D–E and Supplementary Table), consistent with enhanced long-term survival and tumorigenic potential. Additionally, wound-healing assays showed accelerated gap closure in *UCK2*-overexpressing cells, with substantially greater migration at 24 h compared with control groups (Fig. 13F–G and Supplementary Table). These gain-of-function findings directly complement the knockdown results and provide bidirectional genetic evidence that *UCK2* is a critical regulator of proliferative and migratory behavior in HCC.

## Discussion

This integrated analysis across TCGA-LIHC and GEO cohorts nominates a coherent miRNA/mRNA program in HCC centered on an inverse, survival-relevant mir-125b-2-*UCK2* axis, with secondary support from miR-21, and exploratory roles for miR-221 and miR-9-1. By intersecting TargetScan/MiRDB predictions with direction-consistent DEGs, we derived 25 miRNA/mRNA pairs, prioritized seven targets by overall-survival screening (risk: *UCK2*; tumor-suppressive: *CPEB3*,* PIK3R1*,* SUN2*,* CLDN14*,* PDK4*,* OGDHL*) and showed that composite “axes” features stratify prognosis in TCGA and validate in GSE31384. Single-cell communication analysis highlighted epithelial localization of *UCK2* and dense stromal–epithelial signaling wired through fibronectin/laminin and growth-factor circuits (HGF/MET, PDGF). Immune deconvolution suggested a shift away from undifferentiated M0 macrophages with relative increases in polarized macrophage states and selected lymphoid subsets in gene-high strata. Collectively, these results suggest that an ECM/growth-factor circuit couples to nucleoside salvage metabolism via *UCK2* and is modulated by specific miRNAs with plausible therapeutic implications.

Extensive prior work supports the oncogenic roles of miR-21 and miR-221 in HCC and other tumors. In HCC, miR-21 overexpression is repeatedly linked to adverse clinicopathological features and poor survival in meta-analyses and surgical cohorts, while acknowledging some heterogeneity across populations and treatments [35]. Likewise, miR-221 is a canonical oncomiR in liver cancer: it directly represses the CDK inhibitors p27^Kip1 and p57^Kip2, thereby promoting cell-cycle progression; functional silencing of miR-221 restrains HCC growth in preclinical models [36]. By contrast, miR-125b behaves largely as a tumor suppressor in HCC. Its restoration impedes proliferation, glycolysis, and chemoresistance, consistent with our observation that higher miR-125b associates with better OS and inversely tracks target-set activity [37]. miR-9 is context-dependent but frequently pro-metastatic in HCC, where it down-regulates E-cadherin and promotes EMT, invasion, and worse prognosis which is mirroring our directionality at the abundance level, even though cohort-level coherence with its specific supported target set was limited [38].

The nomination of *UCK2* as the central, adverse-risk target is strongly grounded in prior reports. *UCK2*,uridine-cytidine kinase 2, is a rate-limiting enzyme of the pyrimidine salvage pathway that is overexpressed across cancers including HCC [39]. Its high expression correlates with aggressive clinicopathologic features, metastasis and inferior survival [40]. Mechanistically, *UCK2* supports nucleotide supply and, in some settings, intersects with growth-factor signaling; recent studies underscore its metabolic and non-metabolic oncogenic roles and even show HCC growth dependence on *UCK2* under nutrient stress. Our data (higher tumor expression, hypomethylation and adverse survival) extend and consolidate these observations in a multi-omic framework [41].

The six protective targets in our screen align well with literature describing tumor-suppressive functions in liver or related cancers. *CPEB3*, an RNA-binding protein, antagonizes EGFR signaling and suppresses metastatic programs supporting its favorable directionality in our survival analysis [42]. *PIK3R1* (p85α), the class IA PI3K regulatory subunit, exhibits tumor-suppressor properties via restraint of p110 catalytic activity and positive modulation of PTEN [43]. Multiple studies link reduced *PIK3R1* to worse outcomes or hyperactive PI3K signaling in HCC and other tumors, consistent with our observation that higher expression is protective [44]. *SUN2*, a LINC-complex inner nuclear membrane protein, is generally reduced in cancers and associated with better survival when preserved [45]. Although detailed HCC-specific data are still emerging, its tumor-suppressive behavior and mechano-nuclear roles justify the favorable association we observe [46]. *CLDN14*, a tight-junction claudin, is epigenetically silenced in HCC via EZH2 and its loss predicts poor survival; thus, higher *CLDN14* aligning with improved OS in our analysis recapitulates prior clinical observations [47]. *PDK4*, a gatekeeper of pyruvate dehydrogenase, is frequently downregulated in HCC; restoring/maintaining PDK4 constrains lipogenesis and tumor growth, matching our protective signal [48]. Finally, *OGDHL*, an oxidative metabolism enzyme, shows promoter hypermethylation, copy-number loss and reduced expression in HCC [49]. Functional studies indicate it restrains tumor progression, again resonating with our survival-favorable direction [50].

Our enrichment and CellChat analyses converge on ECM and growth-factor circuitry that is well established in HCC biology. The HGF/MET axis drives proliferation, invasion, EMT, and angiogenesis and has long been pursued therapeutically in liver cancer; our pathway-level signals and communication networks that highlight HGF are fully consonant with this literature [51]. In HCC, FN1 overexpression associates with vascular invasion, and laminin-332 promotes adhesion and motility which is consistent with our FN1/LAMININ traffic and a pro-mitogenic matrix milieu [52]. PDGF signaling organizes fibroblasts and perivascular cells in the tumor stroma [53]. MIF is increasingly linked to immunosuppression and poor outcomes in liver and other cancers [54].

The observed reduction of undifferentiated M0 macrophages with relative increases in polarized macrophage states (M1/M2) and selected lymphoid populations coheres with current understanding of macrophage plasticity and its impact on HCC immunity. While M2 polarization is often immunosuppressive, the mixed pattern we observe across cohorts likely reflects dynamic remodeling rather than a unidirectional shift. This is consistent with contemporary macrophage literature in HCC [54].

Clinically, the miR-125b-2/UCK2 axis can be positioned as a biomarker-defined stratification layer for systemic therapy. Contemporary first-line standards for unresectable HCC include immune checkpoint blockade combined with anti-VEGF therapy (e.g., atezolizumab plus bevacizumab) and dual checkpoint strategies such as tremelimumab plus durvalumab, with multi-kinase inhibitors (e.g., sorafenib, lenvatinib) remaining relevant in specific settings and lines of therapy [55]. In this context, a miR-125b-2-low/UCK2-high state plausibly captures tumors with increased proliferative/metabolic demand and ECM/growth-factor wiring. It suggests two actionable directions: (i) combination strategies pairing immune-based regimens with agents targeting growth-factor/MET/VEGFR signaling (given the established role of HGF/c-MET biology in HCC and the clinical activity of multi-target TKIs such as cabozantinib), and (ii) metabolism-informed combinations that exploit nucleotide-salvage dependency [40, 51, 56]. A second implication is treatment resistance: miR-125b family biology has been linked to sorafenib resistance through EMT/stemness-associated programs in HCC which suggest the miR-125b-low strata as candidates for alternative or combination systemic strategies [57]. Finally, UCK2 itself has been known as a predictive biomarker for cytidine analog prodrugs that require UCK2 for activation (e.g., RX-3117 in other tumor types) which raise the hypothesis that UCK2-high HCC could be selectively vulnerable to UCK2-activated nucleoside analog strategies [58].

Although our findings were derived from TCGA-LIHC and externally validated in independent GEO cohorts, all analyses were retrospective and based on publicly available transcriptomic datasets. Limitations include the absence of a prospectively collected clinical cohort with standardized treatment, small ML training sample size, lack of pearson correlation analysis and follow-up limits the immediate translational generalizability of the miR-125b-2/UCK2 axis as a prognostic biomarker. Future studies should evaluate this axis in prospectively annotated, multi-center patient populations to determine direct miRNA-mRNA binding and whether miR-125b-2/UCK2 stratification retains independent prognostic value beyond established clinical staging systems.

## Conclusion

This multi-cohort analysis defines a convergent miR-125b-2/UCK2 regulatory axis in HCC that links epithelial proliferation, nucleotide metabolism and extracellular matrix/growth factor (ECM/GF) signaling. Across independent datasets, miR-125b-2 emerged as the most coherent downregulated regulator, while UCK2 was the dominant adverse prognostic effector, exhibiting tumor overexpression, hypomethylation, and epithelial lineage enrichment. Survival modeling demonstrated that the composite axis score robustly stratifies patients into distinct risk groups, supporting its potential utility for molecular risk assessment beyond conventional clinicopathologic parameters. Clinically, the miR-125b-2/UCK2 axis may serve as a tractable biomarker system for identifying biologically aggressive, metabolically active HCC. Given UCK2’s role in pyrimidine salvage and its association with PI3K/AKT–MAPK and ECM-associated signaling programs, tumors with high UCK2 expression may represent a growth factor–responsive subset with enhanced proliferative and invasive capacity. This raises the possibility that therapeutic strategies targeting nucleotide metabolism could be rationally combined with receptor tyrosine kinase or ECM/GF pathway inhibitors in selected patients. 

## Supplementary Information

Supplementary Material 1.

Supplementary Material 2.

## Data Availability

Bulk and scRNA transcriptomic datasets for training and validation were downloaded from the NCBI Gene Expression Omnibus (GEO) ( https://www.ncbi.nlm.nih.gov/geo/) and TCGA-LIHC ( https://portal.gdc.cancer.gov/projects/TCGA-LIHC). The custom R scripts are available from the corresponding author upon reasonable request.
