# Supplementary material for: Extracellular matrix-growth factor signalling drives the oncogenic mir-125b-2/UCK2 axis in hepatocellular carcinoma
Source: Hereditas. 2026 Apr 2;163:59. doi: 10.1186/s41065-026-00673-y (PMC13169607; doi:10.1186/s41065-026-00673-y)
Supplement: Supplementary file 2 — Supplementary Material 2. [file 41065_2026_673_MOESM2_ESM.docx]

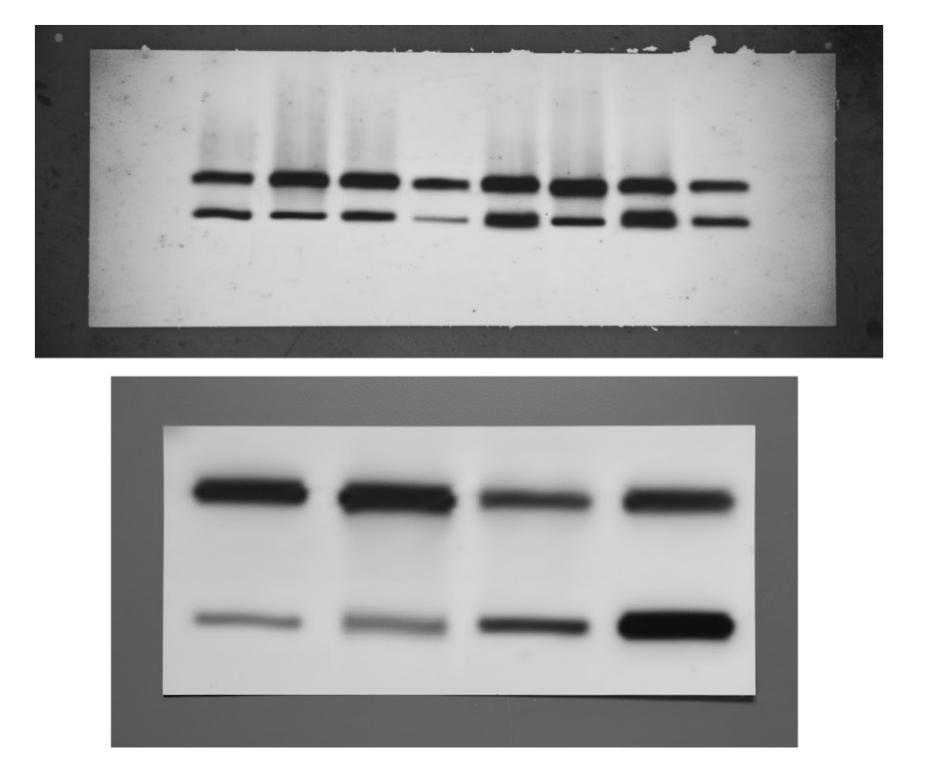


**Supplementary Figure 1: Uncut Western blot bands of *UCK2* and *GAPDH*.**

**
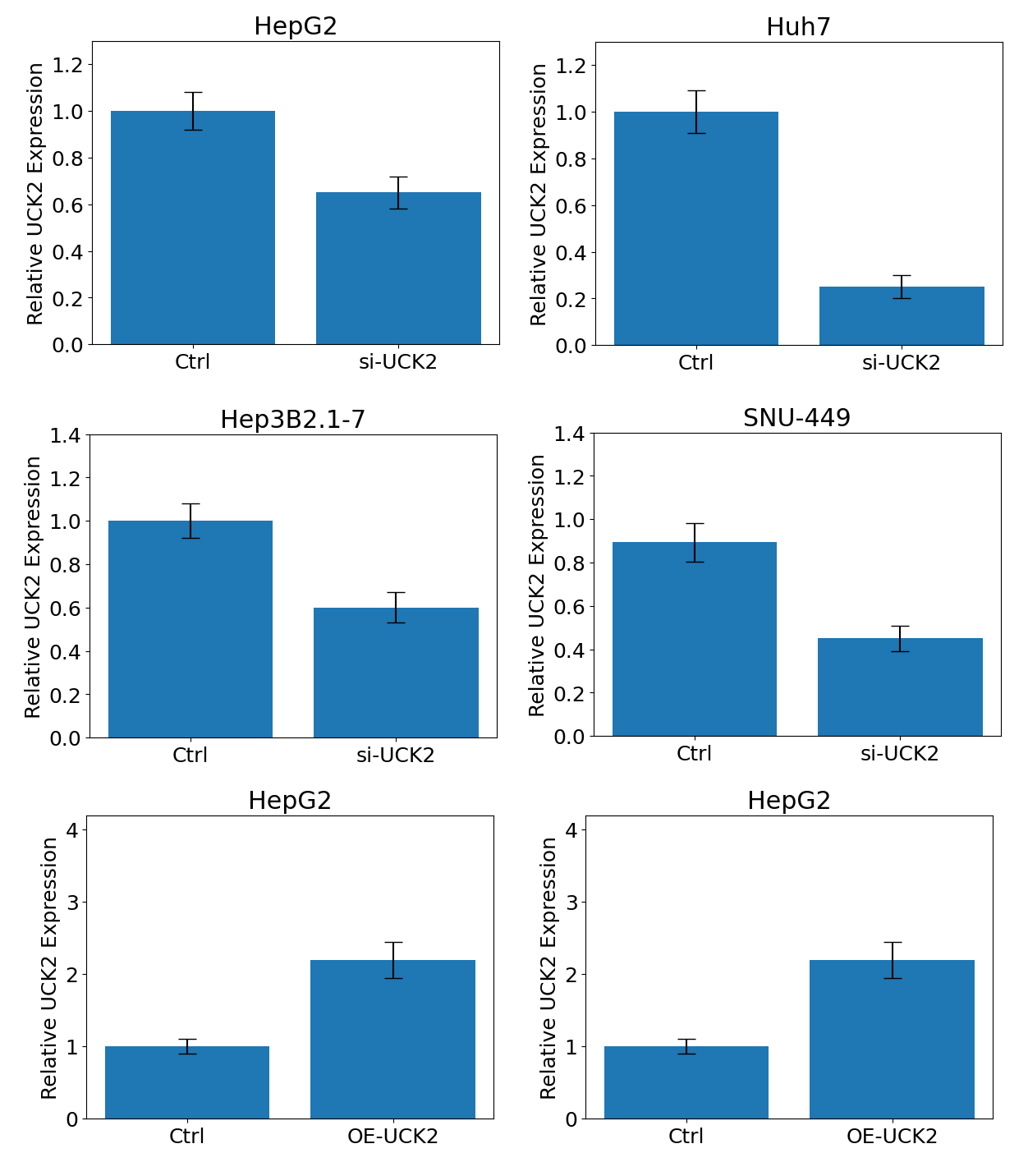
**

**Supplementary Figure 2: Densiometric analysis graphs.**
